# Supplementary material for: Prospective cohort study comparing a triceps-sparing and triceps-detaching approach in total elbow arthroplasty: a protocol
Source: BMJ Open. 2021 May 5;11(5):e046098. doi: 10.1136/bmjopen-2020-046098 (PMC8103376; doi:10.1136/bmjopen-2020-046098)
Supplement: Author's manuscript [file bmjopen-2020-046098.draft_revisions.pdf]

BMJ Open is committed to open peer review. As part of this commitment we make the peer review history of every article we publish publicly available.

When an article is published we post the peer reviewers' comments and the authors' responses online. We also post the versions of the paper that were used during peer review. These are the versions that the peer review comments apply to.

The versions of the paper that follow are the versions that were submitted during the peer review process. They are not the versions of record or the final published versions. They should not be cited or distributed as the published version of this manuscript.

BMJ Open is an open access journal and the full, final, typeset and author-corrected version of record of the manuscript is available on our site with no access controls, subscription charges or pay-per-view fees (http://bmjopen.bmj.com).

If you have any questions on BMJ Open's open peer review process please email info.bmjopen@bmj.com

BMJ Open

A prospective cohort study comparing a triceps-sparing and triceps-detaching approach in total elbow arthroplasty.

Journal:	*BMJ Open*
Manuscript ID	bmjopen-2020-046098
Article Type:	Protocol
Date Submitted by the Author:	22-Oct-2020
Complete List of Authors:	Meijering, Danielle; University Medical Centre Groningen, Department of Orthopedic Surgery<br>Boerboom, Alexander L; University Medical Centre Groningen, Department of Orthopedic Surgery<br>Gerritsma-Bleeker, Carina; Martini Hospital, Department of Orthopaedic Surgery<br>The, Bertram; Amphia Hospital, Department of Orthopedic Surgery<br>van den Bekerom, Michel ; OLVG, Department of Orthopedic Surgery; Vrije Universiteit Amsterdam, Department of Human Movement Sciences, Faculty of Behavioral and Movement Sciences<br>van der Pluijm, Marco; Sint Maartenskliniek, Department of Orthopedic Surgery<br>Vegter, Riemer JK; University of Groningen, Department of Human Movement Sciences<br>Bulstra, Sjoerd ; University Medical Centre Groningen, Department of Orthopedic Surgery<br>Eygendaal, Denise; Amphia Hospital, Department of Orthopedic Surgery; Amsterdam Universitair Medische Centra, Department of Orthopedic Surgery<br>Stevens, Martin; University Medical Centre Groningen, Department of Orthopedic Surgery
Keywords:	Elbow & shoulder < ORTHOPAEDIC & TRAUMA SURGERY, Limb reconstruction < ORTHOPAEDIC & TRAUMA SURGERY, Musculoskeletal disorders < ORTHOPAEDIC & TRAUMA SURGERY, Adult orthopaedics < ORTHOPAEDIC & TRAUMA SURGERY, Orthopaedic & trauma surgery < SURGERY, Clinical trials < THERAPEUTICS

SCHOLARONE™
Manuscripts

I, the Submitting Author has the right to grant and does grant on behalf of all authors of the Work (as defined in the below author licence), an exclusive licence and/or a non-exclusive licence for contributions from authors who are: i) UK Crown employees; ii) where BMJ has agreed a CC-BY licence shall apply, and/or iii) in accordance with the terms applicable for US Federal Government officers or employees acting as part of their official duties; on a worldwide, perpetual, irrevocable, royalty-free basis to BMJ Publishing Group Ltd ("BMJ") its licensees and where the relevant Journal is co-owned by BMJ to the co-owners of the Journal, to publish the Work in this journal and any other BMJ products and to exploit all rights, as set out in our licence.

The Submitting Author accepts and understands that any supply made under these terms is made by BMJ to the Submitting Author unless you are acting as an employee on behalf of your employer or a postgraduate student of an affiliated institution which is paying any applicable article publishing charge ("APC") for Open Access articles. Where the Submitting Author wishes to make the Work available on an Open Access basis (and intends to pay the relevant APC), the terms of reuse of such Open Access shall be governed by a Creative Commons licence – details of these licences and which Creative Commons licence will apply to this Work are set out in our licence referred to above.

Other than as permitted in any relevant BMJ Author's Self Archiving Policies, I confirm this Work has not been accepted for publication elsewhere, is not being considered for publication elsewhere and does not duplicate material already published. I confirm all authors consent to publication of this Work and authorise the granting of this licence.

For peer review only

A prospective cohort study comparing a triceps-sparing and triceps-detaching approach in total elbow arthroplasty.

Authors:

Daniëlle Meijering – MD[1], d.meijering@umcg.nl

Alexander L Boerboom – MD[1], a.l.boerboom@umcg.nl

Carina LE Gerritsma – MD, PhD[2], c.l.e.gerritsma@mzh.nl

Bertram The – MD, PhD[3], bthe@amphia.nl

Michel PJ van den Bekerom – MD, PhD[4,5], m.p.j.vandenbekerom@olvg.nl

Marco van der Pluijm – MD[6], m.vanderpluijm@maartenskliniek.nl

Riemer JK Vegter – PhD[7], r.j.k.vegter@umcg.nl

Sjoerd K Bulstra - MD, PhD[1], s.k.bulstra@umcg.nl

Denise Eygendaal – MD, PhD[3,8], deygendaal@amphia.nl

Martin Stevens – PhD[1], m.stevens@umcg.nl

on behalf of the study group.

Department of Orthopedic Surgery, University Medical Center Groningen, University of Groningen, Groningen, The Netherlands[1].

Department of Orthopedic Surgery, Martini Hospital, Groningen, The Netherlands[2].

Department of Orthopedic Surgery, Amphia Hospital, Breda, The Netherlands[3].

Department of Orthopedic Surgery, OLVG, Amsterdam, The Netherlands[4].

Department of Human Movement Sciences, Faculty of Behavioral and Movement Sciences, Vrije Universiteit Amsterdam, Amsterdam Movement Sciences, Amsterdam, The Netherlands[5].

Department of Orthopedic Surgery, Sint Maartenskliniek, Nijmegen, The Netherlands[6].

Department of Human Movement Sciences, University of Groningen, University Medical Center Groningen, Groningen, The Netherlands[7].

For peer review only - http://bmjopen.bmj.com/site/about/guidelines.xhtml

Department of Orthopedic Surgery, Amsterdam University Medical Centers, Amsterdam, The Netherlands[8].

Corresponding author: D. Meijering – d.meijering@umcg.nl

For peer review only

For peer review only - http://bmjopen.bmj.com/site/about/guidelines.xhtml

Abstract

Background: New surgical approaches have been developed to optimise elbow function after total elbow arthroplasty (TEA). Currently there is no consensus on the best surgical approach. This study aims to investigate the functional outcomes, prosthetic component position and complication rates after a triceps-sparing and a triceps-detaching approach in TEA.

Methods and analysis: A multicentre prospective comparative cohort study will be conducted. All patients with an indication for primary TEA will enrol in either the triceps-sparing or the triceps-detaching cohort. Primary outcome measure is elbow function, specified as fixed flexion deformity (FFD). Secondary outcome parameters are self-reported and objectively measured physical functioning, including triceps force, prosthetic component position in standard radiographs and complications.

Discussion: The successful completion of this study will clarify which surgical approach yields better functional outcomes, better prosthetic component position and lower complication rates in patients with a TEA.

Ethics and dissemination: The Medical Ethics Review Board of University Medical Center Groningen reviewed the study and concluded that it is not clinical research with human subjects as meant in the Medical Research Involving Human Subjects Act (WMO), therefore WMO approval is not needed (METc2019/544).

Trial registration: The trial is registered in the Dutch Trial Register: NTR NL8488.

Keywords: elbow joint, elbow prosthesis, arthroplasty, surgical approach, triceps insufficiency, triceps-sparing, triceps-detaching.

For peer review only - http://bmjopen.bmj.com/site/about/guidelines.xhtml

Strengths and limitations of this study

- This is the first prospective cohort study to compare functional outcomes, prosthetic component position and complication rates following triceps-sparing and triceps-detaching approaches in TEA.
- A limitation of the study is that it is non-blinded and non-randomised.

For peer review only

Background

New surgical approaches have been developed in recent decades to optimise elbow function and reduce complication rates following total elbow arthroplasty (TEA). Several surgical options exist, including a triceps-sparing approach and a triceps-detaching approach. To date, there are no prospective studies comparing triceps-sparing and triceps-detaching approaches, and full insight into the benefits and drawbacks of the two approaches is lacking.

Functional outcomes following TEA can severely deteriorate due to postoperative complications such as triceps insufficiency or long-term complications like aseptic loosening of the prosthesis (1,2). Lenoir et al. showed that functional outcomes are also affected by prosthetic component positioning (3). A triceps sparing approach has recently been advocated because of triceps insufficiency as a complication following triceps-detaching approaches. Another drawback of a triceps-detaching approach is the need to immobilise in a cast, which might impede postoperative elbow function. A triceps-sparing approach makes direct functional treatment is possible, with potentially better functional outcomes.

Several studies already tried to shed light into which surgical approach yields better functional outcomes. In 2015 Dachs et al. (4) conducted a retrospective analysis to compare triceps-sparing and triceps-detaching approaches. They concluded that elbow function, more specifically fixed flexion deformity (FFD), was better in patients following a triceps-sparing approach. They also concluded that triceps-related complications were absent in triceps-sparing approaches. Other authors (5-7) presented similar studies with comparable results, favouring a triceps-sparing approach.

For peer review only - http://bmjopen.bmj.com/site/about/guidelines.xhtml

These authors, however, did not analyse prosthetic component position on radiographs. Considering the fact that the triceps-sparing approach results in less exposure of the articular surface (8), prosthetic component position might be compromised. King et al. (9) already showed that a triceps-sparing approach might lead to a more flexed position of the ulnar component. It is also known that implant malalignment increases loading patterns (10), which might cause polyethylene wear and early loosening of the prosthesis (11).

Hence, there is no consensus on the best surgical approach in TEA. Based on retrospective studies it can be hypothesised that a triceps-sparing approach gives favourable results in terms of functional outcomes and a lower risk of triceps insufficiency. Due to the limited visibility, prosthetic component position may be compromised though.

Aim of this study is therefore to investigate the functional outcomes, prosthetic component position in standard radiographs and complication rates following a triceps-sparing and a triceps-detaching approach in TEA.

For peer review only - http://bmjopen.bmj.com/site/about/guidelines.xhtml

Methods

Study design

A multicentre prospective cohort study will be conducted at University Medical Center Groningen, Martini Hospital Groningen, Amphia Hospital Breda, OLVG Amsterdam and Sint Maartenskliniek Nijmegen. In total 102 patients will be included. The first cohort of 51 patients will be assigned to the triceps-sparing group, the second cohort of 51 patients to the triceps-detaching group. This strategy will provide continuity in the surgical approach for surgeons and avoid surgeon-based inclusion bias.

Recruitment and consent

All adult patients with an indication for primary TEA will be asked to participate in the study. The treating surgeon or a member of the study staff will introduce and explain the study to the patient and answer any questions the patient might have. Patients will receive written information document, and after giving informed consent they will be added to the study database.

Study population

Inclusion criteria: age ≥ 18 years, primary total elbow prosthesis, and ability to participate during the entire follow-up schedule. Exclusion criteria: active infection, total elbow prosthesis surgery in the past (at the ipsilateral side) i.e. revision surgery, previous elbow surgery that influences function of the triceps muscle, other upper extremity injuries to the ipsilateral limb that would compromise postoperative rehabilitation, inability to follow postoperative rehabilitation (due to head injury, dementia, mental

For peer review only - http://bmjopen.bmj.com/site/about/guidelines.xhtml

illness, etc.), insufficient command of the Dutch language, and conversion of surgical technique during the operation.

Intervention

Patients will be placed in the lateral decubitus position. Tranexamic acid 1gr and cefazolin 2gr will be administered. A dorsal skin incision is made. The incision is curved to pass laterallt to the olecranon tip. Full-thickness subcutaneous flaps are developed. The ulnar nerve is located and released. Then depending on the cohort, the specific surgical technique will be performed and prosthetic components will be placed. A tourniquet is inflated only during cementing. All patients will receive a linked type Latitude TEA, without radial head replacement. The radial head will not be resected unless severely damaged.

Once the tourniquet is deflated and range of motion is assessed, the ulnar nerve is returned to its preoperative position or transposed anteriorly in case of tension to the nerve. Refixation of collateral ligaments will be performed whenever possible, and the wound is closed with sutures or staples.

Cefazolin 1gr will be given 8 and 16 hours after initial dose, tranexamic acid 1gr 8 hours after initial dose.

Triceps sparing – cohort 1

After full-thickness skin flaps are developed, medial and lateral windows along the edge of the triceps are created. The triceps attachment remains intact to the olecranon, and

For peer review only - http://bmjopen.bmj.com/site/about/guidelines.xhtml

after release of the collateral ligaments and opening of the joint capsule the joint can be dislocated (12).

Active flexion, extension, pronation and supination are commenced on the first postoperative day. After three weeks patients are allowed to lift 1kg repetitively and 5kg occasionally.

Triceps detaching – cohort 2

A triceps detaching technique as described by Van Gorder et al. (13) will be used. This approach reflects the aponeurosis of the m. triceps downwards, with the base on the olecranon. The underlying muscle is longitudinally split in the midline and elevated. Postoperatively the elbow will be protected by a removable cast in 30° flexion for four weeks, avoiding active extension. Exercises with active flexion and passive extension are allowed, 3 times a day. Thereafter, the elbow will be mobilised without a brace and active triceps training is allowed. After three weeks, patients are allowed to lift 1kg repetitively and 5kg occasionally.

For peer review only

Outcome measures

Demographics

Age, sex, hand dominance, indication for surgery and previous surgery will be registered.

Primary outcome

The primary outcome measure is elbow function, described as fixed flexion deformity (FFD), measured in degrees of flexion using a goniometer.

For peer review only - http://bmjopen.bmj.com/site/about/guidelines.xhtml

Secondary outcomes

Secondary outcomes are self-reported physical functioning, objectively measured physical functioning, prosthetic component position on AP and lateral radiographs and complications.

Self-reported physical functioning

- Elbow function will be measured with the Oxford Elbow Score (OES) (14). The OES consists of three domains; pain, function and social-psychological. Each domain comprises four questions with five response options per question. Each response is scored 0 to 4, with 0 representing greater severity. Scores for each domain are calculated as the sum of each individual item scored within that domain. These scores are then converted to a metric score between 0 and 48, where a lower score represents greater severity. The Dutch language version is considered reliable and valid (15).
- Upper limb function will be assessed using the Quick Disabilities of Arm Shoulder and Hand (Quick-DASH) (16). The DASH gives a score out of 100, where a higher score indicates greater disability. The questionnaire is available in Dutch and is considered reliable and valid (16).
- Health-related quality of life will be measured by the EQ5D-5L (17), a widely used and valid generic instrument to measure health-related quality of life that is validated in the Dutch language (18,19). EQ5D-5L has five dimensions: mobility, self-care, usual activities, pain/discomfort and anxiety/depression.

For peer review only - http://bmjopen.bmj.com/site/about/guidelines.xhtml

Each dimension is divided into five degrees of severity: no problems, slight problems, moderate problems, severe problems and extreme problems or unable to do. Current quality of life must also be identified on the EQ5D-5L Visual Analogue Scale.

- Elbow pain; level will be determined using a 10-point Numeric Rating Scale (NRS). Pain level will be scored during activities and rest.

- Satisfaction; patients are asked whether they are satisfied with their elbow procedure and whether they would recommend it to others. These items are self-constructed and consist of five answer options (Completely agree, Agree, Neutral, Disagree, Completely disagree). In addition, patients in the triceps-detaching cohort are asked about their satisfaction with the removable cast.

Objectively measured physical functioning

- Active and passive range of motion (ROM) (flexion, extension, pronation, supination) will be measured using a goniometer. A systematic review analysing use of a goniometer in elbow measurements showed high intra-rater and interrater reliability of the universal goniometer (20).

- Triceps brachii force will be measured with a MicroFET and expressed in Newtons. Measurements will be with the elbow in 30° flexion as described by Prkic et al. (21). In case of a flexion contracture >30°, measurement will be done in 60° flexion. In case of an acute fracture, preoperative force will be measured contralateralyl. The results of the study by Prkic et al. (21) will then be used to calculate the triceps brachii force on the operated elbow.

For peer review only - http://bmjopen.bmj.com/site/about/guidelines.xhtml

- Triceps brachii function will be measured using the Medical Research Council (MRC) scale.

- Elbow stability will be stated as intact, <10° instability or >10° instability.

- Neurovascular function; motoric and sensory deficits of the ulnar, medial and radial nerves will be tested.

- Paraesthesia of the ulnar nerve will be measured using the Tinel Test.

- Carrying angle; expressed in varus/valgus degrees and measured using a goniometer.

- Swelling of the elbow; stated as none, minimal, some, excessive.

- Pain during palpation will be testing by locating the position of the pain.

Prosthetic component positioning

- Positioning of the prosthesis will be analysed on anteroposterior and lateral radiographs at six weeks and one year follow-up. Flexion, extension, varus and valgus positioning of both the humeral and ulnar components will be measured in degrees, as described by Lenoir et al. (22).

Complications

- Number of complications and type of complication will be registered.

<u>Study procedures</u>

Clinical assessment will be performed at baseline, six months and one year after TEA. At each follow-up visit the surgeon will conduct a physical examination and capture complications, as described previously. Patients will fill out the questionnaires at baseline

For peer review only - http://bmjopen.bmj.com/site/about/guidelines.xhtml

and at six months and one year follow-up. At six weeks and one year follow-up, anteroposterior and lateral radiographs of the elbow will be taken to analyse component position. Results of questionnaires and radiological analysis will be stored digitally in the Research Electronic Data Capture system (Redcap). Physical examination and complications will be documented in medical records, then transported to Redcap.

Sample size calculation

To calculate the sample size, FFD is used as primary outcome measure. To detect 12° difference in elbow function, described as FFD, at one year follow-up, a total of 102 patients are needed in this study. This power is based on earlier research by Dachs et al. that describes FFD after triceps-sparing and triceps-detaching surgical approaches in TEA (4). In this study the difference in FFD was 12° (SD19.6), which was considered clinically relevant, since a functional arc of motion in daily life is described as flexion-extension = 120-30-0 (23). Based on this, a two-sided test with $\alpha = 0{,}05$ and a power of 80%, a sample size of 84 patients is needed. Taking into account a 20% loss of subjects, a total of 102 patients (51 in each group) is needed.

Statistical analysis

Descriptive statistics are used to describe patients' characteristics, clinical outcomes and scores on the questionnaires. Primary outcome of the study is FFD at one year follow-up. Three measurements will be collected, preoperatively and at six months and one year follow-up. The dependency of FFD between the triceps-sparing group and the triceps-detaching group at one year follow-up is our focal interest. To take into account the

For peer review only - http://bmjopen.bmj.com/site/about/guidelines.xhtml

dependencies (the nesting structure of the three measurements nested within patients), a longitudinal multilevel model will be used to analyse the data. This model has a level 1 (measurement) and a level 2 (patient). Relevant covariates such as age, gender and indication for surgery will be controlled for. The results will be considered statistically significant if $p < 0.05$. SPSS statistical software (version 24.0, IBM SPSS, Chicago) will be used.

Ethics and dissemination

The Medical Ethics Review Board of University Medical Center Groningen reviewed the study and concluded that it is not clinical research with human subjects as meant in the Medical Research Involving Human Subjects Act (WMO); therefore WMO approval is not needed (METc2019/544). Eligible patients will be informed about the study and will sign an informed consent form in order to participate. All informed consent forms will be stored in a locked research office and no personal data will be stored digitally in the Redcap system or revealed in any publication or scientific journal.

Considering current evidence, no clear preference exists for either one of the treatment protocols in this study. Both protocols are regularly applied and all surgeons participating in this study are familiar with the two surgical approaches. Management will not differ between patients, except for the surgical approach. Patients will be exposed to radiation from radiographs, but this is part of routine clinical care. No additional radiographs will be taken as part of this study. Patients may be inconvenienced by filling out questionnaires, which takes approximately 15-20 minutes at three different time points, but in most hospitals this too is part of routine clinical care.

For peer review only

For peer review only - http://bmjopen.bmj.com/site/about/guidelines.xhtml

278 Patient and public involvement

279 Patients and public were not directly involved in the development of the research
280 question or in the design of the study.

For peer review only

Discussion

To date, there are no prospective studies comparing triceps-sparing and triceps-detaching approaches in TEA. Based on retrospective studies it is hypothesised that triceps-sparing approaches may lead to better elbow function and a lower risk of triceps insufficiency. However, these studies did not analyse prosthetic component position. Considering the technical nature of the triceps-sparing approach, it may carry a higher risk of component malposition, which can cause excessive loads and could lead to polyethylene wear and early loosening of the prosthesis (11). Both triceps-sparing and triceps-detaching approaches are currently being used (2,24), and persistently high complication rates following TEA are reported (1,2). This stresses the need for a prospective comparative study on the best surgical approach, in order to optimise elbow function and reduce complication rates.

In recent decades, studies have reported an increasing number of TEAs performed globally (25). This is partly due to the changing trend in indications for TEA, from primary and rheumatoid arthritis to acute trauma and posttraumatic deformities (25) and partly due to an aging population. As the incidence of falls and fall-related injury increases with age, a further rise in the numbers of TEAs is expected.

In conclusion, currently there is no consensus on the best surgical approach in TEA and full insight into the benefits and drawbacks of the two approaches is lacking. The successful completion of this study will shed light into which surgical approach, triceps-sparing or a triceps-detaching, results in better functional outcomes, better prosthetic component position and lower complication rates.

For peer review only - http://bmjopen.bmj.com/site/about/guidelines.xhtml

Patient enrolment started in March 2020 and we expect to enrol 50 patients per year. Considering a one-year follow-up, publication of data is expected in 2023.

For peer review only

For peer review only - http://bmjopen.bmj.com/site/about/guidelines.xhtml

Declarations

Ethics approval and consent to participate

The Medical Ethics Review Board of University Medical Center Groningen reviewed the study and concluded that it is not clinical research with human subjects as meant in the Medical Research Involving Human Subjects Act (WMO), therefore WMO approval is not needed (METc2019/544).

Competing interest and funding

Conflict of interest: none related to this manuscript.

D Eygendaal: I disclose the following financial relationships with commercial entities that produce healthcare-related products or services: institutional research support, Matthys; institutional research support, Zimmer-Biomet; institutional research support, Stryker; speaker/teacher for A.O. and IBRA courses; advisory board member for Lima corporates.

Funding: there is no funding source.

Collaborators of the study group

MPJ van den Bekerom, AL Boerboom, SK Bulstra, D Eygendaal, CLE Gerritsma, P Heesterbeek, K Koenraadt, D Meijering, I van Oost, LIF Penning, M van der Pluijm, M Stevens, S Susan, B The, RJK Vegter, S Vorrink, A de Vries.

Authors' contributions

The study is coordinated by DM, ALB, DE, MS. MS will act as principal investigator. DM, ALB, DE, SKB and MS developed the trial and drafted the manuscript. CLEG, BT, MPJB, MP, RJKV revised the manuscript for important intellectual content.

For peer review only

For peer review only - http://bmjopen.bmj.com/site/about/guidelines.xhtml

327 ALB, CLEG, BT, MPJB, MP, LIFP, and DE will participate in patient inclusion.

328 All authors have read and approved the manuscript.

329 Data sharing statement

330 Individual participant data that underlie the results reported in future articles will be

331 available from the corresponding author on reasonable request after deidentification.

332 Acknowledgements

333 The authors thank Dr. R.E. Stewart of UMCG, for his assistance with the statistical

334 analyses.

For peer review only

For peer review only - http://bmjopen.bmj.com/site/about/guidelines.xhtml

References

(1) Welsink CL, Lambers KTA, van Deurzen, D F P, Eygendaal D, van den Bekerom, M P J. Total Elbow Arthroplasty: A Systematic Review. JBJS Rev 2017 July 01;5(7):e4.

(2) Voloshin I, Schippert DW, Kakar S, Kaye EK, Morrey BF. Complications of total elbow replacement: A systematic review. Journal of Shoulder and Elbow Surgery 2011;20(1):158-168.

(3) Lenoir H, Micallef JP, Djerbi I, Waitzenegger T, Lazerges C, Chammas M, et al. Total elbow arthroplasty: Influence of implant positioning on functional outcomes. Orthopaedics & Traumatology: Surgery & Research 2015;101(6):721-727.

(4) Dachs, Robert P., MBChB (UCT), FCS(SA) Orth, Mmed (Orth) UCT, Fleming, Mark A., MBChB (WITS), FCS(SA) Orth, Chivers, David A., MBChB (WITS), Carrara HR, MPH, Du Plessis, Jean-Pierre, MBChB (UCT), FCS(SA) Orth, Vrettos, Basil C., MBChB (UCT), FCS(SA) Orth, FRCS (Eng), Mmed (Orth) UCT, et al. Total elbow arthroplasty: outcomes after triceps-detaching and triceps-sparing approaches. Journal of Shoulder and Elbow Surgery 2015;24(3):339-347.

(5) Studer A, Athwal GS, MacDermid JC, Faber KJ, King GJ. The lateral para-olecranon approach for total elbow arthroplasty. J Hand Surg Am 2013 November 01;38(11):2219-2226.e3.

(6) Large R, Tambe A, Cresswell T, Espag M, Clark DI. Medium-term clinical results of a linked total elbow replacement system. Bone Joint J 2014 October 01;96-B(10):1359-1365.

(7) Solarz MK, Patel MK, Struk AM, Matthias R, King JJ, Wright TW, et al. A Clinical Comparison of Triceps-Sparing and Triceps-Detaching Approaches for Revision Total Elbow Arthroplasty. J Hand Surg Am 2020 January 01;45(1):66.e1-66.e6.

(8) Booker SJ, Smith CD. Triceps on approach for total elbow arthroplasty: worth preserving? A review of approaches for total elbow arthroplasty. Shoulder & Elbow 2017 Apr;9(2):105-111.

(9) King A, Booker SJ, Thomas WJ, Smith CD. Triceps on, alignment off? A comparison of total elbow arthroplasty component positioning with a triceps-on and a triceps-off approach. Ann R Coll Surg Engl 2018 August 16:1-6.

(10) Brownhill JR, Pollock JW, Ferreira LM, Johnson JA, King GJ. The effect of implant linking and ligament integrity on humeral loading of a convertible total elbow arthroplasty. Shoulder & elbow 2019 Feb;11(1):45-52.

For peer review only - http://bmjopen.bmj.com/site/about/guidelines.xhtml

(11) Brinkman JM, de Vos MJ, Eygendaal D. Failure mechanisms in uncemented Kudo type 5 elbow prosthesis in patients with rheumatoid arthritis: 7 of 49 ulnar components revised because of loosening after 2-10 years. Acta Orthop 2007 April 01;78(2):263-270.

(12) Alonso-Lames M. Bilaterotricipital approach to elbow. Its application in the osteosynthesis of supracondylar fractures in yhe humerus in children. Acta Orthop Scand 1972;43:479-90.

(13) VANGORDER GW. SURGICAL APPROACH IN OLD POSTERIOR DISLOCATION OF THE ELBOW. 1932 JBJS: January - Volume 14 - Issue 1 - p 127-143.

(14) Dawson J, Doll H, Boller I, Fitzpatrick R, Little C, Rees J, et al. Comparative responsiveness and minimal change for the Oxford Elbow Score following surgery. Qual Life Res 2008 December 01;17(10):1257-1267.

(15) de Haan J, Goei H, Schep NW, Tuinebreijer WE, Patka P, den Hartog D. The reliability, validity and responsiveness of the Dutch version of the Oxford elbow score. J Orthop Surg Res 2011 July 30;6:39-39.

(16) Veehof MM, Sleegers EJ, van Veldhoven NH, Schuurman AH, van Meeteren NL. Psychometric qualities of the Dutch language version of the Disabilities of the Arm, Shoulder, and Hand questionnaire (DASH-DLV). J Hand Ther 2002 December 01;15(4):347-354.

(17) Versteegh MM, Rowen D, Brazier JE, Stolk EA. Mapping onto Eq-5 D for patients in poor health. Health Qual Life Outcomes 2010 November 26;8:141-141.

(18) Janssen MF, Pickard AS, Golicki D, Gudex C, Niewada M, Scalone L, et al. Measurement properties of the EQ-5D-5L compared to the EQ-5D-3L across eight patient groups: a multi-country study. Qual Life Res 2013 September 01;22(7):1717-1727.

(19) M Versteegh M, M Vermeulen K, M A A Evers, S, de Wit GA, Prenger R, A Stolk E. Dutch Tariff for the Five-Level Version of EQ-5D. Value Health 2016 June 01;19(4):343-352.

(20) van Rijn SF, Zwerus EL, Koenraadt KL, Jacobs WC, van den Bekerom, M P, Eygendaal D. The reliability and validity of goniometric elbow measurements in adults: A systematic review of the literature. Shoulder Elbow 2018 October 01;10(4):274-284.

(21) Prkic A, Viveen J, The B, van Bergen CJ, Koenraadt KL, Eygendaal D. Comparison of isometric triceps brachii force measurement in different elbow positions. J Orthop Surg (Hong Kong) 2018 August 01;26(2):2309499018783907.

For peer review only - http://bmjopen.bmj.com/site/about/guidelines.xhtml

(22) Lenoir H, Micallef JP, Djerbi I, Waitzenegger T, Lazerges C, Chammas M, et al. Total elbow arthroplasty: Influence of implant positioning on functional outcomes. Orthopaedics & Traumatology: Surgery & Research 2015;101(6):721-727.

(23) Morrey BF, Askew LJ, Chao EY. A biomechanical study of normal functional elbow motion. Journal of bone and joint surgery. American volume 1981;63(6):872-877.

(24) Little CP, Graham AJ, Carr AJ. Total elbow arthroplasty: a systematic review of the literature in the English language until the end of 2003. J Bone Joint Surg Br 2005 April 01;87(4):437-444.

(25) Macken AA, Prkic A, Kodde IF, Lans J, Chen NC, Eygendaal D. Global trends in indications for total elbow arthroplasty: a systematic review of national registries. EFORT Open Rev 2020 April 02;5(4):215-220.

(26) Hox J, Van de Schoot R, Matthijsse S. How few countries will do? Comparative survey analysis from a Bayesian perspective. 2012(Surv Res Methods 2012;6:87–93.).

(27) Maas C HJ. Sufficient sample sizes for multilevel modeling. . 2005(Methodology 2005;1: 86–92).

For peer review only

For peer review only - http://bmjopen.bmj.com/site/about/guidelines.xhtml

BMJ Open

A prospective cohort study comparing a triceps-sparing and triceps-detaching approach in total elbow arthroplasty.

Journal:	*BMJ Open*
Manuscript ID	bmjopen-2020-046098.R1
Article Type:	Protocol
Date Submitted by the Author:	22-Mar-2021
Complete List of Authors:	Meijering, Danielle; University Medical Centre Groningen, Department of Orthopedic Surgery<br>Boerboom, Alexander L; University Medical Centre Groningen, Department of Orthopedic Surgery<br>Gerritsma-Bleeker, Carina; Martini Hospital, Department of Orthopaedic Surgery<br>The, Bertram; Amphia Hospital, Department of Orthopedic Surgery<br>van den Bekerom, Michel ; OLVG, Department of Orthopedic Surgery; Vrije Universiteit Amsterdam, Department of Human Movement Sciences, Faculty of Behavioral and Movement Sciences<br>van der Pluijm, Marco; Sint Maartenskliniek, Department of Orthopedic Surgery<br>Vegter, Riemer JK; University of Groningen, Department of Human Movement Sciences<br>Bulstra, Sjoerd ; University Medical Centre Groningen, Department of Orthopedic Surgery<br>Eygendaal, Denise; Amphia Hospital, Department of Orthopedic Surgery; Amsterdam Universitair Medische Centra, Department of Orthopedic Surgery<br>Stevens, Martin; University Medical Centre Groningen, Department of Orthopedic Surgery
**Primary Subject Heading**:	Surgery
Secondary Subject Heading:	Research methods
Keywords:	Elbow & shoulder < ORTHOPAEDIC & TRAUMA SURGERY, Limb reconstruction < ORTHOPAEDIC & TRAUMA SURGERY, Musculoskeletal disorders < ORTHOPAEDIC & TRAUMA SURGERY, Adult orthopaedics < ORTHOPAEDIC & TRAUMA SURGERY, Orthopaedic & trauma surgery < SURGERY, Clinical trials < THERAPEUTICS

SCHOLARONE™ Manuscripts

I, the Submitting Author has the right to grant and does grant on behalf of all authors of the Work (as defined in the below author licence), an exclusive licence and/or a non-exclusive licence for contributions from authors who are: i) UK Crown employees; ii) where BMJ has agreed a CC-BY licence shall apply, and/or iii) in accordance with the terms applicable for US Federal Government officers or employees acting as part of their official duties; on a worldwide, perpetual, irrevocable, royalty-free basis to BMJ Publishing Group Ltd ("BMJ") its licensees and where the relevant Journal is co-owned by BMJ to the co-owners of the Journal, to publish the Work in this journal and any other BMJ products and to exploit all rights, as set out in our licence.

The Submitting Author accepts and understands that any supply made under these terms is made by BMJ to the Submitting Author unless you are acting as an employee on behalf of your employer or a postgraduate student of an affiliated institution which is paying any applicable article publishing charge ("APC") for Open Access articles. Where the Submitting Author wishes to make the Work available on an Open Access basis (and intends to pay the relevant APC), the terms of reuse of such Open Access shall be governed by a Creative Commons licence – details of these licences and which Creative Commons licence will apply to this Work are set out in our licence referred to above.

Other than as permitted in any relevant BMJ Author's Self Archiving Policies, I confirm this Work has not been accepted for publication elsewhere, is not being considered for publication elsewhere and does not duplicate material already published. I confirm all authors consent to publication of this Work and authorise the granting of this licence.

For peer review only

A prospective cohort study comparing a triceps-sparing and triceps-detaching approach in total elbow arthroplasty.

Authors:

Daniëlle Meijering – MD[1], d.meijering@umcg.nl

Alexander L Boerboom – MD[1], a.l.boerboom@umcg.nl

Carina LE Gerritsma – MD, PhD[2], c.l.e.gerritsma@mzh.nl

Bertram The – MD, PhD[3], bthe@amphia.nl

Michel PJ van den Bekerom – MD, PhD[4,5], m.p.j.vandenbekerom@olvg.nl

Marco van der Pluijm – MD[6], m.vanderpluijm@maartenskliniek.nl

Riemer JK Vegter – PhD[7], r.j.k.vegter@umcg.nl

Sjoerd K Bulstra - MD, PhD[1], s.k.bulstra@umcg.nl

Denise Eygendaal – MD, PhD[3,8], deygendaal@amphia.nl

Martin Stevens – PhD[1], m.stevens@umcg.nl

on behalf of the study group.

Department of Orthopedic Surgery, University Medical Center Groningen, University of Groningen, Groningen, The Netherlands[1].

Department of Orthopedic Surgery, Martini Hospital, Groningen, The Netherlands[2].

Department of Orthopedic Surgery, Amphia Hospital, Breda, The Netherlands[3].

Department of Orthopedic Surgery, OLVG, Amsterdam, The Netherlands[4].

Department of Human Movement Sciences, Faculty of Behavioral and Movement Sciences, Vrije Universiteit Amsterdam, Amsterdam Movement Sciences, Amsterdam, The Netherlands[5].

Department of Orthopedic Surgery, Sint Maartenskliniek, Nijmegen, The Netherlands[6].

Department of Human Movement Sciences, University of Groningen, University Medical Center Groningen, Groningen, The Netherlands[7].

For peer review only - http://bmjopen.bmj.com/site/about/guidelines.xhtml

Department of Orthopedic Surgery, Amsterdam University Medical Centers, Amsterdam, The Netherlands[8.]

Corresponding author: D. Meijering – d.meijering@umcg.nl

For peer review only

Abstract

Background: New surgical approaches have been developed to optimise elbow function after total elbow arthroplasty (TEA). Currently there is no consensus on the best surgical approach. This study aims to investigate the functional outcomes, prosthetic component position and complication rates after a triceps-sparing and a triceps-detaching approach in TEA.

Methods and analysis: A multicentre prospective comparative cohort study will be conducted. All patients with an indication for primary TEA will enrol in either the triceps-sparing or the triceps-detaching cohort. Primary outcome measure is elbow function, specified as fixed flexion deformity (FFD). Secondary outcome parameters are self-reported and objectively measured physical functioning, including triceps force, prosthetic component position in standard radiographs and complications.

Discussion: The successful completion of this study will clarify which surgical approach yields better functional outcomes, better prosthetic component position and lower complication rates in patients with a TEA.

Ethics and dissemination: The Medical Ethics Review Board of University Medical Center Groningen reviewed the study and concluded that it is not clinical research with human subjects as meant in the Medical Research Involving Human Subjects Act (WMO), therefore WMO approval is not needed (METc2019/544).

Trial registration: The trial is registered in the Dutch Trial Register: NTR NL8488.

Keywords: elbow joint, elbow prosthesis, arthroplasty, surgical approach, triceps insufficiency, triceps-sparing, triceps-detaching.

For peer review only - http://bmjopen.bmj.com/site/about/guidelines.xhtml

Strengths and limitations of this study

- This is the first prospective cohort study to compare functional outcomes, prosthetic component position and complication rates following triceps-sparing and triceps-detaching approaches in TEA.
- The hospitals participating in this study represent the vast majority (over ±70%) of the TEAs annually performed in the Netherlands.
- A limitation of the study is that it is non-blinded and non-randomised.

For peer review only

For peer review only - http://bmjopen.bmj.com/site/about/guidelines.xhtml

Background

New surgical approaches have been developed in recent decades to optimise elbow function and reduce complication rates following total elbow arthroplasty (TEA). Several surgical options exist, including a triceps-sparing approach and a triceps-detaching approach. To date, there are no prospective studies comparing triceps-sparing and triceps-detaching approaches, and full insight into the benefits and drawbacks of the two approaches is lacking.

Functional outcomes following TEA can severely deteriorate due to postoperative complications such as triceps insufficiency or long-term complications like aseptic loosening of the prosthesis (1,2). Lenoir et al. showed that functional outcomes are also affected by prosthetic component positioning (3). A triceps sparing approach has recently been advocated because of triceps insufficiency as a complication following triceps-detaching approaches. Another drawback of a triceps-detaching approach is the need to immobilise in a cast, which might impede postoperative elbow function. A triceps-sparing approach makes direct functional treatment is possible, with potentially better functional outcomes.

Several studies already tried to shed light into which surgical approach yields better functional outcomes. In 2015 Dachs et al. (4) conducted a retrospective analysis to compare triceps-sparing and triceps-detaching approaches. They concluded that elbow function, more specifically fixed flexion deformity (FFD), was better in patients following a triceps-sparing approach. They also concluded that triceps-related complications were absent in triceps-sparing approaches. Other authors (5-7) presented similar studies with comparable results, favoring a triceps-sparing approach.

For peer review only - http://bmjopen.bmj.com/site/about/guidelines.xhtml

These authors, however, did not analyse prosthetic component position on radiographs. Considering the fact that the triceps-sparing approach results in less exposure of the articular surface (8), prosthetic component position might be compromised. King et al. (9) already showed that a triceps-sparing approach might lead to a more flexed position of the ulnar component. It is also known that implant malalignment increases loading patterns (10), which might cause polyethylene wear and early loosening of the prosthesis (11).

Hence, there is no consensus on the best surgical approach in TEA. Based on retrospective studies it can be hypothesised that a triceps-sparing approach gives favorable results in terms of functional outcomes and a lower risk of triceps insufficiency. Due to the limited visibility, prosthetic component position may be compromised though. Aim of this study is therefore to investigate the functional outcomes, prosthetic component position in standard radiographs and complication rates following a triceps-sparing and a triceps-detaching approach in TEA.

For peer review only

For peer review only - http://bmjopen.bmj.com/site/about/guidelines.xhtml

Methods

Study design

A multicentre prospective cohort study will be conducted at University Medical Center Groningen, Martini Hospital Groningen, Amphia Hospital Breda, OLVG Amsterdam and Sint Maartenskliniek Nijmegen. The hospitals participating in this study represent the vast majority (over ±70%) of the TEAs annually performed in the Netherlands. In total 102 patients will be included. The first cohort of 51 patients will be assigned to the triceps-sparing group, the second cohort of 51 patients to the triceps-detaching group. This strategy will provide continuity in the surgical approach for surgeons and avoid surgeon-based inclusion bias.

Recruitment and consent

All adult patients with an indication for primary TEA will be asked to participate in the study. The treating surgeon or a member of the study staff will introduce and explain the study to the patient and answer any questions the patient might have. Patients will receive written information document, and after giving informed consent they will be added to the study database.

Study population

Inclusion criteria: age ≥ 18 years, primary total elbow prosthesis, and ability to participate during the entire follow-up schedule. Exclusion criteria: active infection, total elbow prosthesis surgery in the past (at the ipsilateral side) i.e. revision surgery, previous elbow surgery that influences function of the triceps muscle, other upper extremity

For peer review only - http://bmjopen.bmj.com/site/about/guidelines.xhtml

injuries to the ipsilateral limb that would compromise postoperative rehabilitation, inability to follow postoperative rehabilitation (due to head injury, dementia, mental illness, etc.), insufficient command of the Dutch language, and conversion of surgical technique during the operation.

Intervention

Patients will be placed in the lateral decubitus position. Tranexamic acid 1gr and cefazolin 2gr will be administered. A dorsal skin incision is made. The incision is curved to pass lateral to the olecranon tip. Full-thickness subcutaneous flaps are developed. The ulnar nerve is located and released. Then depending on the cohort, the specific surgical technique will be performed and prosthetic components will be placed. A tourniquet is inflated only during cementing. All patients will receive a linked type Latitude TEA, without radial head replacement. The radial head will not be resected unless severely damaged.

Once the tourniquet is deflated and range of motion is assessed, the ulnar nerve is returned to its preoperative position or transposed anteriorly in case of tension to the nerve. Refixation of collateral ligaments will be performed whenever possible, and the wound is closed with sutures or staples.

Cefazolin 1gr will be given 8 and 16 hours after initial dose, tranexamic acid 1gr 8 hours after initial dose.

Triceps sparing – cohort 1

For peer review only - http://bmjopen.bmj.com/site/about/guidelines.xhtml

After full-thickness skin flaps are developed, medial and lateral windows along the edge of the triceps are created. The triceps attachment remains intact to the olecranon, and after release of the collateral ligaments and opening of the joint capsule the joint can be dislocated (12).

Active flexion, extension, pronation and supination are commenced on the first postoperative day. After three weeks patients are allowed to lift 1kg repetitively and 5kg occasionally.

Triceps detaching – cohort 2

A triceps detaching technique as described by Van Gorder et al. (13) will be used. This approach reflects the aponeurosis of the m. triceps downwards, with the base on the olecranon. The underlying muscle is longitudinally split in the midline and elevated. Postoperatively the elbow will be protected by a removable cast in 30° flexion for four weeks, avoiding active extension. This time span was chosen arbitrary, to take the post-operative healing of the triceps into account. Unfortunately, evidence for the best aftertreatment following a triceps-detaching approach is unavailable and therefore this protocol is historically rooted in our daily practice. Exercises with active flexion and passive extension are allowed, 3 times a day. Thereafter, the elbow will be mobilised without a brace and active triceps training is allowed. After three weeks, patients are allowed to lift 1kg repetitively and 5kg occasionally.

<u>Outcome measures</u>

Demographics

For peer review only - http://bmjopen.bmj.com/site/about/guidelines.xhtml

Age, sex, hand dominance, indication for surgery and previous surgery will be registered.

Primary outcome

The primary outcome measure is elbow function, described as fixed flexion deformity (FFD), measured in degrees of flexion using a goniometer.

Secondary outcomes

Secondary outcomes are self-reported physical functioning, objectively measured physical functioning, prosthetic component position on AP and lateral radiographs and complications.

Self-reported physical functioning

- Elbow function will be measured with the Oxford Elbow Score (OES) (14). The OES consists of three domains; pain, function and social-psychological. Each domain comprises four questions with five response options per question. Each response is scored 0 to 4, with 0 representing greater severity. Scores for each domain are calculated as the sum of each individual item scored within that domain. These scores are then converted to a metric score between 0 and 48, where a lower score represents greater severity. The Dutch language version is considered reliable and valid (15).
- Upper limb function will be assessed using the Quick Disabilities of Arm Shoulder and Hand (Quick-DASH) (16). The DASH gives a score out of 100, where a higher score indicates greater disability. The questionnaire is available in Dutch and is considered reliable and valid (16).

For peer review only - http://bmjopen.bmj.com/site/about/guidelines.xhtml

- Health-related quality of life will be measured by the EQ5D-5L (17), a widely used and valid generic instrument to measure health-related quality of life that is validated in the Dutch language (18,19). EQ5D-5L has five dimensions: mobility, self-care, usual activities, pain/discomfort and anxiety/depression.

Each dimension is divided into five degrees of severity: no problems, slight problems, moderate problems, severe problems and extreme problems or unable to do. Current quality of life must also be identified on the EQ5D-5L Visual Analogue Scale.

- Elbow pain; level will be determined using a 10-point Numeric Rating Scale (NRS). Pain level will be scored during activities and rest.

- Satisfaction; patients are asked whether they are satisfied with their elbow procedure and whether they would recommend it to others. These items are self-constructed and consist of five answer options (Completely agree, Agree, Neutral, Disagree, Completely disagree). In addition, patients in the triceps-detaching cohort are asked about their satisfaction with the removable cast.

Objectively measured physical functioning

- Active and passive range of motion (ROM) (flexion, extension, pronation, supination) will be measured using a goniometer. A systematic review analysing use of a goniometer in elbow measurements showed high intra-rater and interrater reliability of the universal goniometer (20).

- Triceps brachii force will be measured with a MicroFET and expressed in Newtons. Measurements will be with the elbow in 30° flexion as described by Prkic et al. (21). In case of a flexion contracture >30°, measurement will be done in 60° flexion. In case of an

For peer review only - http://bmjopen.bmj.com/site/about/guidelines.xhtml

acute fracture, preoperative force will be measured contralateral. The results of the study by Prkic et al. (21) will then be used to calculate the triceps brachii force on the operated elbow.

- Triceps brachii function will be measured using the Medical Research Council (MRC) scale.
- Elbow stability will be stated as intact, <10° instability or >10° instability.
- Neurovascular function; motoric and sensory deficits of the ulnar, medial and radial nerves will be tested. Neurovascular function will be stated as "intact", "sensory deficit", "motoric deficit", "both sensory and motoric deficit".
- Paraesthesia of the ulnar nerve will be measured using the Tinel Test.
- Carrying angle; expressed in varus/valgus degrees and measured using a goniometer.
- Swelling of the elbow; stated as none, minimal, some, excessive.
- Pain during palpation will be testing by locating the position of the pain.

Prosthetic component positioning

- Positioning of the prosthesis will be analysed on anteroposterior and lateral radiographs at six weeks and one year follow-up. Flexion, extension, varus and valgus positioning of both the humeral and ulnar components will be measured in degrees, as described by Lenoir et al. (22).

Complications

- Number of complications and type of complication will be registered.

For peer review only - http://bmjopen.bmj.com/site/about/guidelines.xhtml

For peer review only

Study procedures

Clinical assessment will be performed at baseline, six months and one year after TEA (fig 1). At each follow-up visit the surgeon will conduct a physical examination and capture complications, as described previously. Patients will fill out the questionnaires at baseline and at six months and one year follow-up. At six weeks and one year follow-up, anteroposterior and lateral radiographs of the elbow will be taken to analyse component position. Results of questionnaires and radiological analysis will be stored digitally in the Research Electronic Data Capture system (Redcap). Physical examination and complications will be documented in medical records, then transported to Redcap. Data analysis will be done by an independent researcher.

Sample size calculation

To calculate the sample size, FFD is used as primary outcome measure. To detect 12° difference in elbow function, described as FFD, at one year follow-up, a total of 102 patients are needed in this study. This power is based on earlier research by Dachs et al. that describes FFD after triceps-sparing and triceps-detaching surgical approaches in TEA (4). In this study the difference in FFD was 12° (SD19.6), which was considered clinically relevant, since a functional arc of motion in daily life is described as flexion-extension = 120-30-0 (23). Based on this, a two-sided test with $\alpha = 0,05$ and a power of 80%, a sample size of 84 patients is needed. Taking into account a 20% loss of subjects, a total of 102 patients (51 in each group) is needed.

Statistical analysis

For peer review only - http://bmjopen.bmj.com/site/about/guidelines.xhtml

Descriptive statistics are used to describe patients' characteristics, clinical outcomes and scores on the questionnaires. Means and standard deviations (SDs) will be used for continuous variables, or percentages for categorical variables. Primary outcome of the study is FFD at one year follow-up. Three measurements will be collected, preoperatively and at six months and one year follow-up. The dependency of FFD between the triceps-sparing group and the triceps-detaching group at one year follow-up is our focal interest. To take into account the dependencies (the nesting structure of the three measurements nested within patients), a longitudinal multilevel model will be used to analyse the data. This model has a level 1 (measurement) and a level 2 (patient). Relevant covariates such as age, gender and indication for surgery will be controlled for. Under the assumption of missing data being missing at random, the missing data will be imputed. The results will be considered statistically significant if $p < 0.05$. SPSS statistical software (version 24.0, IBM SPSS, Chicago) will be used.

Ethics and dissemination

The Medical Ethics Review Board of University Medical Center Groningen reviewed the study and concluded that it is not clinical research with human subjects as meant in the Medical Research Involving Human Subjects Act (WMO); therefore WMO approval is not needed (METc2019/544). Eligible patients will be informed about the study and will sign an informed consent form in order to participate. All informed consent forms will be stored in a locked research office and no personal data will be stored digitally in the Redcap system or revealed in any publication or scientific journal.

For peer review only - http://bmjopen.bmj.com/site/about/guidelines.xhtml

Considering current evidence, no clear preference exists for either one of the treatment protocols in this study. Both protocols are regularly applied and all surgeons participating in this study are familiar with the two surgical approaches. Management will not differ between patients, except for the surgical approach. Patients will be exposed to radiation from radiographs, but this is part of routine clinical care. No additional radiographs will be taken as part of this study. Patients may be inconvenienced by filling out questionnaires, which takes approximately 15-20 minutes at three different time points, but in most hospitals this too is part of routine clinical care.

Patient and public involvement

Patients and public were not directly involved in the development of the research question or in the design of the study.

For peer review only

Discussion

To date, there are no prospective studies comparing triceps-sparing and triceps-detaching approaches in TEA. Based on retrospective studies it is hypothesised that triceps-sparing approaches may lead to better elbow function and a lower risk of triceps insufficiency. However, these studies did not analyse prosthetic component position. Considering the technical nature of the triceps-sparing approach, it may carry a higher risk of component malposition, which can cause excessive loads and could lead to polyethylene wear and early loosening of the prosthesis (11). Both triceps-sparing and triceps-detaching approaches are currently being used (2,24), and persistently high complication rates following TEA are reported (1,2). This stresses the need for a prospective comparative study on the best surgical approach, in order to optimise elbow function and reduce complication rates.

The decision to perform a prospective cohort study, instead of a randomized controlled trial (RCT), has been made due to two main reasons. First, we aim to include all Dutch patients requiring a total elbow prosthesis, since numbers of TEAs performed in the Netherlands are low. However our past experience is that a substantial part of the patients is not willing to participate in a RCT. For that we decided to run a cohort study in which in fact usual care is evaluated. By using this design our experience is that more patients are willing to participate. Which is important because the number of TEAs is already quite low. Second, a frequent switch of surgical technique is not desirable, as would be the case in a RCT. Therefore, our study design will provide continuity in the surgical approach for surgeons and for the paramedical care providers (i.e. fysiotherapist, nurse) during a certain period.

For peer review only - http://bmjopen.bmj.com/site/about/guidelines.xhtml

314 In recent decades, studies have reported an increasing number of TEAs performed globally (25). This is partly due to the changing trend in indications for TEA, from primary and rheumatoid arthritis to acute trauma and posttraumatic deformities (25) and partly due to an aging population. As the incidence of falls and fall-related injury increases with age, a further rise in the numbers of TEAs is expected.

319 In conclusion, currently there is no consensus on the best surgical approach in TEA and full insight into the benefits and drawbacks of the two approaches is lacking. The successful completion of this study will shed light into which surgical approach, triceps-sparing or a triceps-detaching, results in better functional outcomes, better prosthetic component position and lower complication rates.

324 Patient enrolment started in March 2020 and we expect to enrol 50 patients per year. Considering a one-year follow-up, publication of data is expected in 2023.

For peer review only

For peer review only - http://bmjopen.bmj.com/site/about/guidelines.xhtml

Declarations

Ethics approval and consent to participate

The Medical Ethics Review Board of University Medical Center Groningen reviewed the study and concluded that it is not clinical research with human subjects as meant in the Medical Research Involving Human Subjects Act (WMO), therefore WMO approval is not needed (METc2019/544).

Competing interest and funding

Conflict of interest: none related to this manuscript.

D Eygendaal: I disclose the following financial relationships with commercial entities that produce healthcare-related products or services: institutional research support, Matthys; institutional research support, Zimmer-Biomet; institutional research support, Stryker; speaker/teacher for A.O. and IBRA courses; advisory board member for Lima corporates.

Funding: there is no funding source.

Collaborators of the study group

MPJ van den Bekerom, AL Boerboom, SK Bulstra, D Eygendaal, CLE Gerritsma, P Heesterbeek, K Koenraadt, D Meijering, I van Oost, LIF Penning, M van der Pluijm, M Stevens, S Susan, B The, RJK Vegter, S Vorrink, A de Vries.

Authors' contributions

The study is coordinated by DM, ALB, DE, MS. MS will act as principal investigator. DM, ALB, DE, SKB and MS developed the trial and drafted the manuscript. CLEG, BT, MPJB, MP, RJKV revised the manuscript for important intellectual content.

For peer review only - http://bmjopen.bmj.com/site/about/guidelines.xhtml

ALB, CLEG, BT, MPJB, MP, LIFP, and DE will participate in patient inclusion.

All authors have read and approved the manuscript.

Data sharing statement

Individual participant data that underlie the results reported in future articles will be available from the corresponding author on reasonable request after deidentification.

Acknowledgements

The authors thank Dr. R.E. Stewart of UMCG, for his assistance with the statistical analyses.

For peer review only

References

(1) Welsink CL, Lambers KTA, van Deurzen, D F P, Eygendaal D, van den Bekerom, M P J. Total Elbow Arthroplasty: A Systematic Review. JBJS Rev 2017 July 01;5(7):e4.

(2) Voloshin I, Schippert DW, Kakar S, Kaye EK, Morrey BF. Complications of total elbow replacement: A systematic review. Journal of Shoulder and Elbow Surgery 2011;20(1):158-168.

(3) Lenoir H, Micallef JP, Djerbi I, Waitzenegger T, Lazerges C, Chammas M, et al. Total elbow arthroplasty: Influence of implant positioning on functional outcomes. Orthopaedics & Traumatology: Surgery & Research 2015;101(6):721-727.

(4) Dachs, Robert P., MBChB (UCT), FCS(SA) Orth, Mmed (Orth) UCT, Fleming, Mark A., MBChB (WITS), FCS(SA) Orth, Chivers, David A., MBChB (WITS), Carrara HR, MPH, Du Plessis, Jean-Pierre, MBChB (UCT), FCS(SA) Orth, Vrettos, Basil C., MBChB (UCT), FCS(SA) Orth, FRCS (Eng), Mmed (Orth) UCT, et al. Total elbow arthroplasty: outcomes after triceps-detaching and triceps-sparing approaches. Journal of Shoulder and Elbow Surgery 2015;24(3):339-347.

(5) Studer A, Athwal GS, MacDermid JC, Faber KJ, King GJ. The lateral para-olecranon approach for total elbow arthroplasty. J Hand Surg Am 2013 November 01;38(11):2219-2226.e3.

(6) Large R, Tambe A, Cresswell T, Espag M, Clark DI. Medium-term clinical results of a linked total elbow replacement system. Bone Joint J 2014 October 01;96-B(10):1359-1365.

(7) Solarz MK, Patel MK, Struk AM, Matthias R, King JJ, Wright TW, et al. A Clinical Comparison of Triceps-Sparing and Triceps-Detaching Approaches for Revision Total Elbow Arthroplasty. J Hand Surg Am 2020 January 01;45(1):66.e1-66.e6.

(8) Booker SJ, Smith CD. Triceps on approach for total elbow arthroplasty: worth preserving? A review of approaches for total elbow arthroplasty. Shoulder & Elbow 2017 Apr;9(2):105-111.

(9) King A, Booker SJ, Thomas WJ, Smith CD. Triceps on, alignment off? A comparison of total elbow arthroplasty component positioning with a triceps-on and a triceps-off approach. Ann R Coll Surg Engl 2018 August 16:1-6.

(10) Brownhill JR, Pollock JW, Ferreira LM, Johnson JA, King GJ. The effect of implant linking and ligament integrity on humeral loading of a convertible total elbow arthroplasty. Shoulder & elbow 2019 Feb;11(1):45-52.

For peer review only

For peer review only - http://bmjopen.bmj.com/site/about/guidelines.xhtml

(11) Brinkman JM, de Vos MJ, Eygendaal D. Failure mechanisms in uncemented Kudo type 5 elbow prosthesis in patients with rheumatoid arthritis: 7 of 49 ulnar components revised because of loosening after 2-10 years. Acta Orthop 2007 April 01;78(2):263-270.

(12) Alonso-Lames M. Bilaterotricipital approach to elbow. Its application in the osteosynthesis of supracondylar fractures in yhe humerus in children. Acta Orthop Scand 1972;43:479-90.

(13) VANGORDER GW. SURGICAL APPROACH IN OLD POSTERIOR DISLOCATION OF THE ELBOW. 1932 JBJS: January - Volume 14 - Issue 1 - p 127-143.

(14) Dawson J, Doll H, Boller I, Fitzpatrick R, Little C, Rees J, et al. Comparative responsiveness and minimal change for the Oxford Elbow Score following surgery. Qual Life Res 2008 December 01;17(10):1257-1267.

(15) de Haan J, Goei H, Schep NW, Tuinebreijer WE, Patka P, den Hartog D. The reliability, validity and responsiveness of the Dutch version of the Oxford elbow score. J Orthop Surg Res 2011 July 30;6:39-39.

(16) Veehof MM, Sleegers EJ, van Veldhoven NH, Schuurman AH, van Meeteren NL. Psychometric qualities of the Dutch language version of the Disabilities of the Arm, Shoulder, and Hand questionnaire (DASH-DLV). J Hand Ther 2002 December 01;15(4):347-354.

(17) Versteegh MM, Rowen D, Brazier JE, Stolk EA. Mapping onto Eq-5 D for patients in poor health. Health Qual Life Outcomes 2010 November 26;8:141-141.

(18) Janssen MF, Pickard AS, Golicki D, Gudex C, Niewada M, Scalone L, et al. Measurement properties of the EQ-5D-5L compared to the EQ-5D-3L across eight patient groups: a multi-country study. Qual Life Res 2013 September 01;22(7):1717-1727.

(19) M Versteegh M, M Vermeulen K, M A A Evers, S, de Wit GA, Prenger R, A Stolk E. Dutch Tariff for the Five-Level Version of EQ-5D. Value Health 2016 June 01;19(4):343-352.

(20) van Rijn SF, Zwerus EL, Koenraadt KL, Jacobs WC, van den Bekerom, M P, Eygendaal D. The reliability and validity of goniometric elbow measurements in adults: A systematic review of the literature. Shoulder Elbow 2018 October 01;10(4):274-284.

(21) Prkic A, Viveen J, The B, van Bergen CJ, Koenraadt KL, Eygendaal D. Comparison of isometric triceps brachii force measurement in different elbow positions. J Orthop Surg (Hong Kong) 2018 August 01;26(2):2309499018783907.

For peer review only - http://bmjopen.bmj.com/site/about/guidelines.xhtml

(22) Lenoir H, Micallef JP, Djerbi I, Waitzenegger T, Lazerges C, Chammas M, et al. Total elbow arthroplasty: Influence of implant positioning on functional outcomes. Orthopaedics & Traumatology: Surgery & Research 2015;101(6):721-727.

(23) Morrey BF, Askew LJ, Chao EY. A biomechanical study of normal functional elbow motion. Journal of bone and joint surgery. American volume 1981;63(6):872-877.

(24) Little CP, Graham AJ, Carr AJ. Total elbow arthroplasty: a systematic review of the literature in the English language until the end of 2003. J Bone Joint Surg Br 2005 April 01;87(4):437-444.

(25) Macken AA, Prkic A, Kodde IF, Lans J, Chen NC, Eygendaal D. Global trends in indications for total elbow arthroplasty: a systematic review of national registries. EFORT Open Rev 2020 April 02;5(4):215-220.

For peer review only

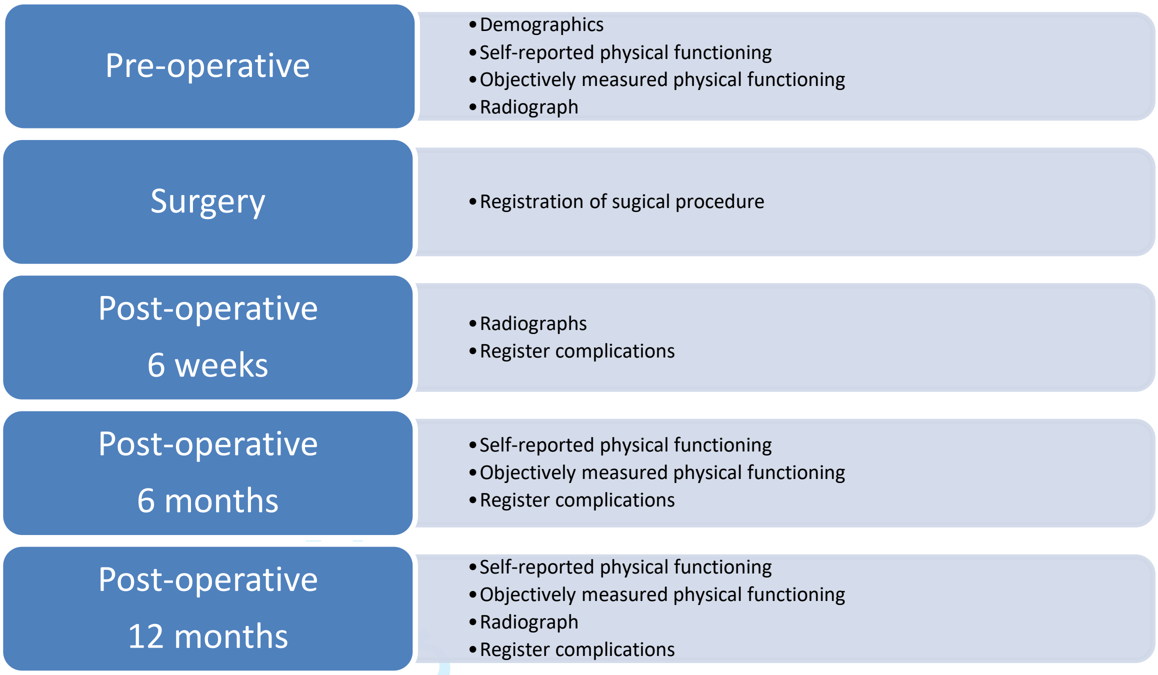

Figure 1. Study procedures

STROBE Statement—Checklist of items that should be included in reports of ***cohort studies***

	Item No	Recommendation	Line
**Title and abstract**	1	(*a*) Indicate the study's design with a commonly used term in the title or the abstract	36
		(*b*) Provide in the abstract an informative and balanced summary of what was done and what was found	36-41
**Introduction**			
Background/rationale	2	Explain the scientific background and rationale for the investigation being reported	60-88
Objectives	3	State specific objectives, including any prespecified hypotheses	89-96
**Methods**			
Study design	4	Present key elements of study design early in the paper	98-104
Setting	5	Describe the setting, locations, and relevant dates, including periods of recruitment, exposure, follow-up, and data collection	99-111 and 228-236
Participants	6	(*a*) Give the eligibility criteria, and the sources and methods of selection of participants. Describe methods of follow-up	114-121
		(*b*) For matched studies, give matching criteria and number of exposed and unexposed	NA
Variables	7	Clearly define all outcomes, exposures, predictors, potential confounders, and effect modifiers. Give diagnostic criteria, if applicable	158-226
Data sources/ measurement	8*	For each variable of interest, give sources of data and details of methods of assessment (measurement). Describe comparability of assessment methods if there is more than one group	158-226
Bias	9	Describe any efforts to address potential sources of bias	100-101, 103-104, 236-237
Study size	10	Explain how the study size was arrived at	238-247
Quantitative variables	11	Explain how quantitative variables were handled in the analyses. If applicable, describe which groupings were chosen and why	249
Statistical methods	12	(*a*) Describe all statistical methods, including those used to control for confounding	250-260
		(*b*) Describe any methods used to examine subgroups and interactions	250-260
		(*c*) Explain how missing data were addressed	260-261
		(*d*) If applicable, explain how loss to follow-up was addressed	NA
		(*e*) Describe any sensitivity analyses	NA
**Results**			
Participants	13*	(a) Report numbers of individuals at each stage of study—eg numbers potentially eligible, examined for eligibility, confirmed eligible, included in the study, completing follow-up, and analysed	NA
		(b) Give reasons for non-participation at each stage	NA
		(c) Consider use of a flow diagram	NA
Descriptive data	14*	(a) Give characteristics of study participants (eg demographic, clinical, social) and information on exposures and potential confounders	NA
		(b) Indicate number of participants with missing data for each variable of interest	NA

For peer review only - http://bmjopen.bmj.com/site/about/guidelines.xhtml

		(c) Summarise follow-up time (eg, average and total amount)	NA
Outcome data	15*	Report numbers of outcome events or summary measures over time	NA
Main results	16	(*a*) Give unadjusted estimates and, if applicable, confounder-adjusted estimates and their precision (eg, 95% confidence interval). Make clear which confounders were adjusted for and why they were included	NA
		(*b*) Report category boundaries when continuous variables were categorized	NA
		(*c*) If relevant, consider translating estimates of relative risk into absolute risk for a meaningful time period	NA
Other analyses	17	Report other analyses done—eg analyses of subgroups and interactions, and sensitivity analyses	NA
**Discussion**			
Key results	18	Summarise key results with reference to study objectives	NA
Limitations	19	Discuss limitations of the study, taking into account sources of potential bias or imprecision. Discuss both direction and magnitude of any potential bias	57
Interpretation	20	Give a cautious overall interpretation of results considering objectives, limitations, multiplicity of analyses, results from similar studies, and other relevant evidence	282-297
Generalisability	21	Discuss the generalisability (external validity) of the study results	298-302
**Other information**			
Funding	22	Give the source of funding and the role of the funders for the present study and, if applicable, for the original study on which the present article is based	NA

*Give information separately for exposed and unexposed groups.

Note: An Explanation and Elaboration article discusses each checklist item and gives methodological background and published examples of transparent reporting. The STROBE checklist is best used in conjunction with this article (freely available on the Web sites of PLoS Medicine at http://www.plosmedicine.org/, Annals of Internal Medicine at http://www.annals.org/, and Epidemiology at http://www.epidem.com/). Information on the STROBE Initiative is available at http://www.strobe-statement.org.

For peer review only

BMJ Open

A prospective cohort study comparing a triceps-sparing and triceps-detaching approach in total elbow arthroplasty: a protocol.

Journal:	*BMJ Open*
Manuscript ID	bmjopen-2020-046098.R2
Article Type:	Protocol
Date Submitted by the Author:	09-Apr-2021
Complete List of Authors:	Meijering, Danielle; University Medical Centre Groningen, Department of Orthopedic Surgery<br>Boerboom, Alexander L; University Medical Centre Groningen, Department of Orthopedic Surgery<br>Gerritsma-Bleeker, Carina; Martini Hospital, Department of Orthopaedic Surgery<br>The, Bertram; Amphia Hospital, Department of Orthopedic Surgery<br>van den Bekerom, Michel ; OLVG, Department of Orthopedic Surgery; Vrije Universiteit Amsterdam, Department of Human Movement Sciences, Faculty of Behavioral and Movement Sciences<br>van der Pluijm, Marco; Sint Maartenskliniek, Department of Orthopedic Surgery<br>Vegter, Riemer JK; University of Groningen, Department of Human Movement Sciences<br>Bulstra, Sjoerd ; University Medical Centre Groningen, Department of Orthopedic Surgery<br>Eygendaal, Denise; Amphia Hospital, Department of Orthopedic Surgery; Amsterdam Universitair Medische Centra, Department of Orthopedic Surgery<br>Stevens, Martin; University Medical Centre Groningen, Department of Orthopedic Surgery
**Primary Subject Heading**:	Surgery
Secondary Subject Heading:	Research methods
Keywords:	Elbow & shoulder < ORTHOPAEDIC & TRAUMA SURGERY, Limb reconstruction < ORTHOPAEDIC & TRAUMA SURGERY, Musculoskeletal disorders < ORTHOPAEDIC & TRAUMA SURGERY, Adult orthopaedics < ORTHOPAEDIC & TRAUMA SURGERY, Orthopaedic & trauma surgery < SURGERY, Clinical trials < THERAPEUTICS

SCHOLARONE™
Manuscripts

I, the Submitting Author has the right to grant and does grant on behalf of all authors of the Work (as defined in the below author licence), an exclusive licence and/or a non-exclusive licence for contributions from authors who are: i) UK Crown employees; ii) where BMJ has agreed a CC-BY licence shall apply, and/or iii) in accordance with the terms applicable for US Federal Government officers or employees acting as part of their official duties; on a worldwide, perpetual, irrevocable, royalty-free basis to BMJ Publishing Group Ltd ("BMJ") its licensees and where the relevant Journal is co-owned by BMJ to the co-owners of the Journal, to publish the Work in this journal and any other BMJ products and to exploit all rights, as set out in our licence.

The Submitting Author accepts and understands that any supply made under these terms is made by BMJ to the Submitting Author unless you are acting as an employee on behalf of your employer or a postgraduate student of an affiliated institution which is paying any applicable article publishing charge ("APC") for Open Access articles. Where the Submitting Author wishes to make the Work available on an Open Access basis (and intends to pay the relevant APC), the terms of reuse of such Open Access shall be governed by a Creative Commons licence – details of these licences and which Creative Commons licence will apply to this Work are set out in our licence referred to above.

Other than as permitted in any relevant BMJ Author's Self Archiving Policies, I confirm this Work has not been accepted for publication elsewhere, is not being considered for publication elsewhere and does not duplicate material already published. I confirm all authors consent to publication of this Work and authorise the granting of this licence.

For peer review only

A prospective cohort study comparing a triceps-sparing and triceps-detaching approach in total elbow arthroplasty: a protocol.

Authors:

Daniëlle Meijering – MD[1], d.meijering@umcg.nl

Alexander L Boerboom – MD[1], a.l.boerboom@umcg.nl

Carina LE Gerritsma – MD, PhD[2], c.l.e.gerritsma@mzh.nl

Bertram The – MD, PhD[3], bthe@amphia.nl

Michel PJ van den Bekerom – MD, PhD[4,5], m.p.j.vandenbekerom@olvg.nl

Marco van der Pluijm – MD[6], m.vanderpluijm@maartenskliniek.nl

Riemer JK Vegter – PhD[7], r.j.k.vegter@umcg.nl

Sjoerd K Bulstra - MD, PhD[1], s.k.bulstra@umcg.nl

Denise Eygendaal – MD, PhD[3,8], deygendaal@amphia.nl

Martin Stevens – PhD[1], m.stevens@umcg.nl

on behalf of the study group.

Department of Orthopedic Surgery, University Medical Center Groningen, University of Groningen, Groningen, The Netherlands[1].

Department of Orthopedic Surgery, Martini Hospital, Groningen, The Netherlands[2].

Department of Orthopedic Surgery, Amphia Hospital, Breda, The Netherlands[3].

Department of Orthopedic Surgery, OLVG, Amsterdam, The Netherlands[4].

Department of Human Movement Sciences, Faculty of Behavioral and Movement Sciences, Vrije Universiteit Amsterdam, Amsterdam Movement Sciences, Amsterdam, The Netherlands[5].

Department of Orthopedic Surgery, Sint Maartenskliniek, Nijmegen, The Netherlands[6].

Department of Human Movement Sciences, University of Groningen, University Medical Center Groningen, Groningen, The Netherlands[7].

For peer review only - http://bmjopen.bmj.com/site/about/guidelines.xhtml

Department of Orthopedic Surgery, Amsterdam University Medical Centers, Amsterdam, The Netherlands[8].

Corresponding author: D. Meijering – d.meijering@umcg.nl

For peer review only

Abstract

Background: New surgical approaches have been developed to optimise elbow function after total elbow arthroplasty (TEA). Currently there is no consensus on the best surgical approach. This study aims to investigate the functional outcomes, prosthetic component position and complication rates after a triceps-sparing and a triceps-detaching approach in TEA.

Methods and analysis: A multicentre prospective comparative cohort study will be conducted. All patients with an indication for primary TEA will enrol in either the triceps-sparing or the triceps-detaching cohort. Primary outcome measure is elbow function, specified as fixed flexion deformity (FFD). Secondary outcome parameters are self-reported and objectively measured physical functioning, including triceps force, prosthetic component position in standard radiographs and complications.

Discussion: The successful completion of this study will clarify which surgical approach yields better functional outcomes, better prosthetic component position and lower complication rates in patients with a TEA.

Ethics and dissemination: The Medical Ethics Review Board of University Medical Center Groningen reviewed the study and concluded that it is not clinical research with human subjects as meant in the Medical Research Involving Human Subjects Act (WMO), therefore WMO approval is not needed (METc2019/544).

Trial registration: The trial is registered in the Dutch Trial Register: NTR NL8488.

Keywords: elbow joint, elbow prosthesis, arthroplasty, surgical approach, triceps insufficiency, triceps-sparing, triceps-detaching.

For peer review only - http://bmjopen.bmj.com/site/about/guidelines.xhtml

Strengths and limitations of this study

- This is the first prospective cohort study to compare functional outcomes, prosthetic component position and complication rates following triceps-sparing and triceps-detaching approaches in TEA.
- The hospitals participating in this study represent the vast majority (over ±70%) of the TEAs annually performed in the Netherlands.
- A limitation of the study is that it is non-blinded and non-randomised.

For peer review only - http://bmjopen.bmj.com/site/about/guidelines.xhtml

Background

New surgical approaches have been developed in recent decades to optimise elbow function and reduce complication rates following total elbow arthroplasty (TEA). Several surgical options exist, including a triceps-sparing approach and a triceps-detaching approach. To date, there are no prospective studies comparing triceps-sparing and triceps-detaching approaches, and full insight into the benefits and drawbacks of the two approaches is lacking.

Functional outcomes following TEA can severely deteriorate due to postoperative complications such as triceps insufficiency or long-term complications like aseptic loosening of the prosthesis (1,2). Lenoir et al. showed that functional outcomes are also affected by prosthetic component positioning (3). A triceps sparing approach has recently been advocated because of triceps insufficiency as a complication following triceps-detaching approaches. Another drawback of a triceps-detaching approach is the need to immobilise in a cast, which might impede postoperative elbow function. A triceps-sparing approach makes direct functional treatment is possible, with potentially better functional outcomes.

Several studies already tried to shed light into which surgical approach yields better functional outcomes. In 2015 Dachs et al. (4) conducted a retrospective analysis to compare triceps-sparing and triceps-detaching approaches. They concluded that elbow function, more specifically fixed flexion deformity (FFD), was better in patients following a triceps-sparing approach. They also concluded that triceps-related complications were absent in triceps-sparing approaches. Other authors (5-7) presented similar studies with comparable results, favoring a triceps-sparing approach.

For peer review only - http://bmjopen.bmj.com/site/about/guidelines.xhtml

These authors, however, did not analyse prosthetic component position on radiographs. Considering the fact that the triceps-sparing approach results in less exposure of the articular surface (8), prosthetic component position might be compromised. King et al. (9) already showed that a triceps-sparing approach might lead to a more flexed position of the ulnar component. It is also known that implant malalignment increases loading patterns (10), which might cause polyethylene wear and early loosening of the prosthesis (11).

Hence, there is no consensus on the best surgical approach in TEA. Based on retrospective studies it can be hypothesised that a triceps-sparing approach gives favorable results in terms of functional outcomes and a lower risk of triceps insufficiency. Due to the limited visibility, prosthetic component position may be compromised though. Aim of this study is therefore to investigate the functional outcomes, prosthetic component position in standard radiographs and complication rates following a triceps-sparing and a triceps-detaching approach in TEA.

For peer review only - http://bmjopen.bmj.com/site/about/guidelines.xhtml

Methods

Study design

A multicentre prospective cohort study will be conducted at University Medical Center Groningen, Martini Hospital Groningen, Amphia Hospital Breda, OLVG Amsterdam and Sint Maartenskliniek Nijmegen. The hospitals participating in this study represent the vast majority (over ±70%) of the TEAs annually performed in the Netherlands. In total 102 patients will be included. The first cohort of 51 patients will be assigned to the triceps-sparing group, the second cohort of 51 patients to the triceps-detaching group. This strategy will provide continuity in the surgical approach for surgeons and avoid surgeon-based inclusion bias.

Recruitment and consent

All adult patients with an indication for primary TEA will be asked to participate in the study. The treating surgeon or a member of the study staff will introduce and explain the study to the patient and answer any questions the patient might have. Patients will receive written information document, and after giving informed consent they will be added to the study database.

Study population

Inclusion criteria: age ≥ 18 years, primary total elbow prosthesis, and ability to participate during the entire follow-up schedule. Exclusion criteria: active infection, total elbow prosthesis surgery in the past (at the ipsilateral side) i.e. revision surgery, previous elbow surgery that influences function of the triceps muscle, other upper extremity

For peer review only - http://bmjopen.bmj.com/site/about/guidelines.xhtml

injuries to the ipsilateral limb that would compromise postoperative rehabilitation, inability to follow postoperative rehabilitation (due to head injury, dementia, mental illness, etc.), insufficient command of the Dutch language, and conversion of surgical technique during the operation.

Intervention

Patients will be placed in the lateral decubitus position. Tranexamic acid 1gr and cefazolin 2gr will be administered. A dorsal skin incision is made. The incision is curved to pass lateral to the olecranon tip. Full-thickness subcutaneous flaps are developed. The ulnar nerve is located and released. Then depending on the cohort, the specific surgical technique will be performed and prosthetic components will be placed. A tourniquet is inflated only during cementing. All patients will receive a linked type Latitude TEA, without radial head replacement. The radial head will not be resected unless severely damaged.

Once the tourniquet is deflated and range of motion is assessed, the ulnar nerve is returned to its preoperative position or transposed anteriorly in case of tension to the nerve. Refixation of collateral ligaments will be performed whenever possible, and the wound is closed with sutures or staples.

Cefazolin 1gr will be given 8 and 16 hours after initial dose, tranexamic acid 1gr 8 hours after initial dose.

Triceps sparing – cohort 1

For peer review only - http://bmjopen.bmj.com/site/about/guidelines.xhtml

After full-thickness skin flaps are developed, medial and lateral windows along the edge of the triceps are created. The triceps attachment remains intact to the olecranon, and after release of the collateral ligaments and opening of the joint capsule the joint can be dislocated (12).

Active flexion, extension, pronation and supination are commenced on the first postoperative day. After three weeks patients are allowed to lift 1kg repetitively and 5kg occasionally.

Triceps detaching – cohort 2

A triceps detaching technique as described by Van Gorder et al. (13) will be used. This approach reflects the aponeurosis of the m. triceps downwards, with the base on the olecranon. The underlying muscle is longitudinally split in the midline and elevated. Postoperatively the elbow will be protected by a removable cast in 30° flexion for four weeks, avoiding active extension. This time span was chosen arbitrary, to take the post-operative healing of the triceps into account. Unfortunately, evidence for the best aftertreatment following a triceps-detaching approach is unavailable and therefore this protocol is historically rooted in our daily practice. Exercises with active flexion and passive extension are allowed, 3 times a day. Thereafter, the elbow will be mobilised without a brace and active triceps training is allowed. After three weeks, patients are allowed to lift 1kg repetitively and 5kg occasionally.

Outcome measures

Demographics

For peer review only - http://bmjopen.bmj.com/site/about/guidelines.xhtml

Age, sex, hand dominance, indication for surgery and previous surgery will be registered.

Primary outcome

The primary outcome measure is elbow function, described as fixed flexion deformity (FFD), measured in degrees of flexion using a goniometer.

Secondary outcomes

Secondary outcomes are self-reported physical functioning, objectively measured physical functioning, prosthetic component position on AP and lateral radiographs and complications.

Self-reported physical functioning

- Elbow function will be measured with the Oxford Elbow Score (OES) (14). The OES consists of three domains; pain, function and social-psychological. Each domain comprises four questions with five response options per question. Each response is scored 0 to 4, with 0 representing greater severity. Scores for each domain are calculated as the sum of each individual item scored within that domain. These scores are then converted to a metric score between 0 and 48, where a lower score represents greater severity. The Dutch language version is considered reliable and valid (15).
- Upper limb function will be assessed using the Quick Disabilities of Arm Shoulder and Hand (Quick-DASH) (16). The DASH gives a score out of 100, where a higher score indicates greater disability. The questionnaire is available in Dutch and is considered reliable and valid (16).

For peer review only - http://bmjopen.bmj.com/site/about/guidelines.xhtml

- Health-related quality of life will be measured by the EQ5D-5L (17), a widely used and valid generic instrument to measure health-related quality of life that is validated in the Dutch language (18,19). EQ5D-5L has five dimensions: mobility, self-care, usual activities, pain/discomfort and anxiety/depression.

Each dimension is divided into five degrees of severity: no problems, slight problems, moderate problems, severe problems and extreme problems or unable to do. Current quality of life must also be identified on the EQ5D-5L Visual Analogue Scale.

- Elbow pain; level will be determined using a 10-point Numeric Rating Scale (NRS). Pain level will be scored during activities and rest.

- Satisfaction; patients are asked whether they are satisfied with their elbow procedure and whether they would recommend it to others. These items are self-constructed and consist of five answer options (Completely agree, Agree, Neutral, Disagree, Completely disagree). In addition, patients in the triceps-detaching cohort are asked about their satisfaction with the removable cast.

Objectively measured physical functioning

- Active and passive range of motion (ROM) (flexion, extension, pronation, supination) will be measured using a goniometer. A systematic review analysing use of a goniometer in elbow measurements showed high intra-rater and interrater reliability of the universal goniometer (20).

- Triceps brachii force will be measured with a MicroFET and expressed in Newtons. Measurements will be with the elbow in 30° flexion as described by Prkic et al. (21). In case of a flexion contracture >30°, measurement will be done in 60° flexion. In case of an

For peer review only - http://bmjopen.bmj.com/site/about/guidelines.xhtml

acute fracture, preoperative force will be measured contralateral. The results of the study by Prkic et al. (21) will then be used to calculate the triceps brachii force on the operated elbow.

- Triceps brachii function will be measured using the Medical Research Council (MRC) scale.
- Elbow stability will be stated as intact, <10° instability or >10° instability.
- Neurovascular function; motoric and sensory deficits of the ulnar, medial and radial nerves will be tested. Neurovascular function will be stated as "intact", "sensory deficit", "motoric deficit", "both sensory and motoric deficit".
- Paraesthesia of the ulnar nerve will be measured using the Tinel Test.
- Carrying angle; expressed in varus/valgus degrees and measured using a goniometer.
- Swelling of the elbow; stated as none, minimal, some, excessive.
- Pain during palpation will be testing by locating the position of the pain.

Prosthetic component positioning

- Positioning of the prosthesis will be analysed on anteroposterior and lateral radiographs at six weeks and one year follow-up. Flexion, extension, varus and valgus positioning of both the humeral and ulnar components will be measured in degrees, as described by Lenoir et al. (22).

Complications

- Number of complications and type of complication will be registered.

For peer review only - http://bmjopen.bmj.com/site/about/guidelines.xhtml

Study procedures

Clinical assessment will be performed at baseline, six months and one year after TEA (fig 1). At each follow-up visit the surgeon will conduct a physical examination and capture complications, as described previously. Patients will fill out the questionnaires at baseline and at six months and one year follow-up. At six weeks and one year follow-up, anteroposterior and lateral radiographs of the elbow will be taken to analyse component position. Results of questionnaires and radiological analysis will be stored digitally in the Research Electronic Data Capture system (Redcap). Physical examination and complications will be documented in medical records, then transported to Redcap. Data analysis will be done by an independent researcher.

Sample size calculation

To calculate the sample size, FFD is used as primary outcome measure. To detect 12° difference in elbow function, described as FFD, at one year follow-up, a total of 102 patients are needed in this study. This power is based on earlier research by Dachs et al. that describes FFD after triceps-sparing and triceps-detaching surgical approaches in TEA (4). In this study the difference in FFD was 12° (SD19.6), which was considered clinically relevant, since a functional arc of motion in daily life is described as flexion-extension = 120-30-0 (23). Based on this, a two-sided test with $\alpha = 0{,}05$ and a power of 80%, a sample size of 84 patients is needed. Taking into account a 20% loss of subjects, a total of 102 patients (51 in each group) is needed.

Statistical analysis

For peer review only - http://bmjopen.bmj.com/site/about/guidelines.xhtml

Descriptive statistics are used to describe patients' characteristics, clinical outcomes and scores on the questionnaires. Means and standard deviations (SDs) will be used for continuous variables, or percentages for categorical variables. Primary outcome of the study is FFD at one year follow-up. Three measurements will be collected, preoperatively and at six months and one year follow-up. The dependency of FFD between the triceps-sparing group and the triceps-detaching group at one year follow-up is our focal interest. To take into account the dependencies (the nesting structure of the three measurements nested within patients), a longitudinal multilevel model will be used to analyse the data. This model has a level 1 (measurement) and a level 2 (patient). Relevant covariates such as age, gender and indication for surgery will be controlled for. Under the assumption of missing data being missing at random, the missing data will be imputed. The results will be considered statistically significant if $p < 0.05$. SPSS statistical software (version 24.0, IBM SPSS, Chicago) will be used.

Ethics and dissemination

The Medical Ethics Review Board of University Medical Center Groningen reviewed the study and concluded that it is not clinical research with human subjects as meant in the Medical Research Involving Human Subjects Act (WMO); therefore WMO approval is not needed (METc2019/544). Eligible patients will be informed about the study and will sign an informed consent form in order to participate. All informed consent forms will be stored in a locked research office and no personal data will be stored digitally in the Redcap system or revealed in any publication or scientific journal.

For peer review only - http://bmjopen.bmj.com/site/about/guidelines.xhtml

Considering current evidence, no clear preference exists for either one of the treatment protocols in this study. Both protocols are regularly applied and all surgeons participating in this study are familiar with the two surgical approaches. Management will not differ between patients, except for the surgical approach. Patients will be exposed to radiation from radiographs, but this is part of routine clinical care. No additional radiographs will be taken as part of this study. Patients may be inconvenienced by filling out questionnaires, which takes approximately 15-20 minutes at three different time points, but in most hospitals this too is part of routine clinical care.

Patient and public involvement

Patients and public were not directly involved in the development of the research question or in the design of the study.

For peer review only - http://bmjopen.bmj.com/site/about/guidelines.xhtml

Discussion

To date, there are no prospective studies comparing triceps-sparing and triceps-detaching approaches in TEA. Based on retrospective studies it is hypothesised that triceps-sparing approaches may lead to better elbow function and a lower risk of triceps insufficiency. However, these studies did not analyse prosthetic component position. Considering the technical nature of the triceps-sparing approach, it may carry a higher risk of component malposition, which can cause excessive loads and could lead to polyethylene wear and early loosening of the prosthesis (11). Both triceps-sparing and triceps-detaching approaches are currently being used (2,24), and persistently high complication rates following TEA are reported (1,2). This stresses the need for a prospective comparative study on the best surgical approach, in order to optimise elbow function and reduce complication rates.

The decision to perform a prospective cohort study, instead of a randomized controlled trial (RCT), has been made due to two main reasons. First, we aim to include all Dutch patients requiring a total elbow prosthesis, since numbers of TEAs performed in the Netherlands are low. However our past experience is that a substantial part of the patients is not willing to participate in a RCT. For that we decided to run a cohort study in which in fact usual care is evaluated. By using this design our experience is that more patients are willing to participate. Which is important because the number of TEAs is already quite low. Second, a frequent switch of surgical technique is not desirable, as would be the case in a RCT. Therefore, our study design will provide continuity in the surgical approach for surgeons and for the paramedical care providers (i.e. physiotherapist, nurse) during a certain period.

For peer review only - http://bmjopen.bmj.com/site/about/guidelines.xhtml

In recent decades, studies have reported an increasing number of TEAs performed globally (25). This is partly due to the changing trend in indications for TEA, from primary and rheumatoid arthritis to acute trauma and posttraumatic deformities (25) and partly due to an aging population. As the incidence of falls and fall-related injury increases with age, a further rise in the numbers of TEAs is expected.

In conclusion, currently there is no consensus on the best surgical approach in TEA and full insight into the benefits and drawbacks of the two approaches is lacking. The successful completion of this study will shed light into which surgical approach, triceps-sparing or a triceps-detaching, results in better functional outcomes, better prosthetic component position and lower complication rates.

Patient enrolment started in March 2020 and we expect to enrol 50 patients per year. Considering a one-year follow-up, publication of data is expected in 2023.

For peer review only

For peer review only - http://bmjopen.bmj.com/site/about/guidelines.xhtml

Declarations

Ethics approval and consent to participate

The Medical Ethics Review Board of University Medical Center Groningen reviewed the study and concluded that it is not clinical research with human subjects as meant in the Medical Research Involving Human Subjects Act (WMO), therefore WMO approval is not needed (METc2019/544).

Competing interest and funding

Conflict of interest: none related to this manuscript.

D Eygendaal: I disclose the following financial relationships with commercial entities that produce healthcare-related products or services: institutional research support, Matthys; institutional research support, Zimmer-Biomet; institutional research support, Stryker; speaker/teacher for A.O. and IBRA courses; advisory board member for Lima corporates. None are related to this manuscript.

Funding: there is no funding source.

Collaborators of the study group

MPJ van den Bekerom, AL Boerboom, SK Bulstra, D Eygendaal, CLE Gerritsma, P Heesterbeek, K Koenraadt, D Meijering, I van Oost, LIF Penning, M van der Pluijm, M Stevens, S Susan, B The, RJK Vegter, S Vorrink, A de Vries.

Authors' contributions

The study is coordinated by DM, ALB, DE, MS. MS will act as principal investigator. DM, ALB, DE, SKB and MS developed the trial and drafted the manuscript. CLEG, BT, MPJB, MP, RJKV revised the manuscript for important intellectual content.

For peer review only

348 ALB, CLEG, BT, MPJB, MP, LIFP, and DE will participate in patient inclusion.

349 All authors have read and approved the manuscript.

350 Data sharing statement

351 Individual participant data that underlie the results reported in future articles will be

352 available from the corresponding author on reasonable request after deidentification.

353 Acknowledgements

354 The authors thank Dr. R.E. Stewart of UMCG, for his assistance with the statistical

355 analyses.

For peer review only

For peer review only - http://bmjopen.bmj.com/site/about/guidelines.xhtml

References

(1) Welsink CL, Lambers KTA, van Deurzen, D F P, Eygendaal D, van den Bekerom, M P J. Total Elbow Arthroplasty: A Systematic Review. JBJS Rev 2017 July 01;5(7):e4.

(2) Voloshin I, Schippert DW, Kakar S, Kaye EK, Morrey BF. Complications of total elbow replacement: A systematic review. Journal of Shoulder and Elbow Surgery 2011;20(1):158-168.

(3) Lenoir H, Micallef JP, Djerbi I, Waitzenegger T, Lazerges C, Chammas M, et al. Total elbow arthroplasty: Influence of implant positioning on functional outcomes. Orthopaedics & Traumatology: Surgery & Research 2015;101(6):721-727.

(4) Dachs, Robert P., MBChB (UCT), FCS(SA) Orth, Mmed (Orth) UCT, Fleming, Mark A., MBChB (WITS), FCS(SA) Orth, Chivers, David A., MBChB (WITS), Carrara HR, MPH, Du Plessis, Jean-Pierre, MBChB (UCT), FCS(SA) Orth, Vrettos, Basil C., MBChB (UCT), FCS(SA) Orth, FRCS (Eng), Mmed (Orth) UCT, et al. Total elbow arthroplasty: outcomes after triceps-detaching and triceps-sparing approaches. Journal of Shoulder and Elbow Surgery 2015;24(3):339-347.

(5) Studer A, Athwal GS, MacDermid JC, Faber KJ, King GJ. The lateral para-olecranon approach for total elbow arthroplasty. J Hand Surg Am 2013 November 01;38(11):2219-2226.e3.

(6) Large R, Tambe A, Cresswell T, Espag M, Clark DI. Medium-term clinical results of a linked total elbow replacement system. Bone Joint J 2014 October 01;96-B(10):1359-1365.

(7) Solarz MK, Patel MK, Struk AM, Matthias R, King JJ, Wright TW, et al. A Clinical Comparison of Triceps-Sparing and Triceps-Detaching Approaches for Revision Total Elbow Arthroplasty. J Hand Surg Am 2020 January 01;45(1):66.e1-66.e6.

(8) Booker SJ, Smith CD. Triceps on approach for total elbow arthroplasty: worth preserving? A review of approaches for total elbow arthroplasty. Shoulder & Elbow 2017 Apr;9(2):105-111.

(9) King A, Booker SJ, Thomas WJ, Smith CD. Triceps on, alignment off? A comparison of total elbow arthroplasty component positioning with a triceps-on and a triceps-off approach. Ann R Coll Surg Engl 2018 August 16:1-6.

(10) Brownhill JR, Pollock JW, Ferreira LM, Johnson JA, King GJ. The effect of implant linking and ligament integrity on humeral loading of a convertible total elbow arthroplasty. Shoulder & elbow 2019 Feb;11(1):45-52.

For peer review only - http://bmjopen.bmj.com/site/about/guidelines.xhtml

(11) Brinkman JM, de Vos MJ, Eygendaal D. Failure mechanisms in uncemented Kudo type 5 elbow prosthesis in patients with rheumatoid arthritis: 7 of 49 ulnar components revised because of loosening after 2-10 years. Acta Orthop 2007 April 01;78(2):263-270.

(12) Alonso-Lames M. Bilaterotricipital approach to elbow. Its application in the osteosynthesis of supracondylar fractures in yhe humerus in children. Acta Orthop Scand 1972;43:479-90.

(13) VANGORDER GW. SURGICAL APPROACH IN OLD POSTERIOR DISLOCATION OF THE ELBOW. 1932 JBJS: January - Volume 14 - Issue 1 - p 127-143.

(14) Dawson J, Doll H, Boller I, Fitzpatrick R, Little C, Rees J, et al. Comparative responsiveness and minimal change for the Oxford Elbow Score following surgery. Qual Life Res 2008 December 01;17(10):1257-1267.

(15) de Haan J, Goei H, Schep NW, Tuinebreijer WE, Patka P, den Hartog D. The reliability, validity and responsiveness of the Dutch version of the Oxford elbow score. J Orthop Surg Res 2011 July 30;6:39-39.

(16) Veehof MM, Sleegers EJ, van Veldhoven NH, Schuurman AH, van Meeteren NL. Psychometric qualities of the Dutch language version of the Disabilities of the Arm, Shoulder, and Hand questionnaire (DASH-DLV). J Hand Ther 2002 December 01;15(4):347-354.

(17) Versteegh MM, Rowen D, Brazier JE, Stolk EA. Mapping onto Eq-5 D for patients in poor health. Health Qual Life Outcomes 2010 November 26;8:141-141.

(18) Janssen MF, Pickard AS, Golicki D, Gudex C, Niewada M, Scalone L, et al. Measurement properties of the EQ-5D-5L compared to the EQ-5D-3L across eight patient groups: a multi-country study. Qual Life Res 2013 September 01;22(7):1717-1727.

(19) M Versteegh M, M Vermeulen K, M A A Evers, S, de Wit GA, Prenger R, A Stolk E. Dutch Tariff for the Five-Level Version of EQ-5D. Value Health 2016 June 01;19(4):343-352.

(20) van Rijn SF, Zwerus EL, Koenraadt KL, Jacobs WC, van den Bekerom, M P, Eygendaal D. The reliability and validity of goniometric elbow measurements in adults: A systematic review of the literature. Shoulder Elbow 2018 October 01;10(4):274-284.

(21) Prkic A, Viveen J, The B, van Bergen CJ, Koenraadt KL, Eygendaal D. Comparison of isometric triceps brachii force measurement in different elbow positions. J Orthop Surg (Hong Kong) 2018 August 01;26(2):2309499018783907.

For peer review only - http://bmjopen.bmj.com/site/about/guidelines.xhtml

(22) Lenoir H, Micallef JP, Djerbi I, Waitzenegger T, Lazerges C, Chammas M, et al. Total elbow arthroplasty: Influence of implant positioning on functional outcomes. Orthopaedics & Traumatology: Surgery & Research 2015;101(6):721-727.

(23) Morrey BF, Askew LJ, Chao EY. A biomechanical study of normal functional elbow motion. Journal of bone and joint surgery. American volume 1981;63(6):872-877.

(24) Little CP, Graham AJ, Carr AJ. Total elbow arthroplasty: a systematic review of the literature in the English language until the end of 2003. J Bone Joint Surg Br 2005 April 01;87(4):437-444.

(25) Macken AA, Prkic A, Kodde IF, Lans J, Chen NC, Eygendaal D. Global trends in indications for total elbow arthroplasty: a systematic review of national registries. EFORT Open Rev 2020 April 02;5(4):215-220.

For peer review only

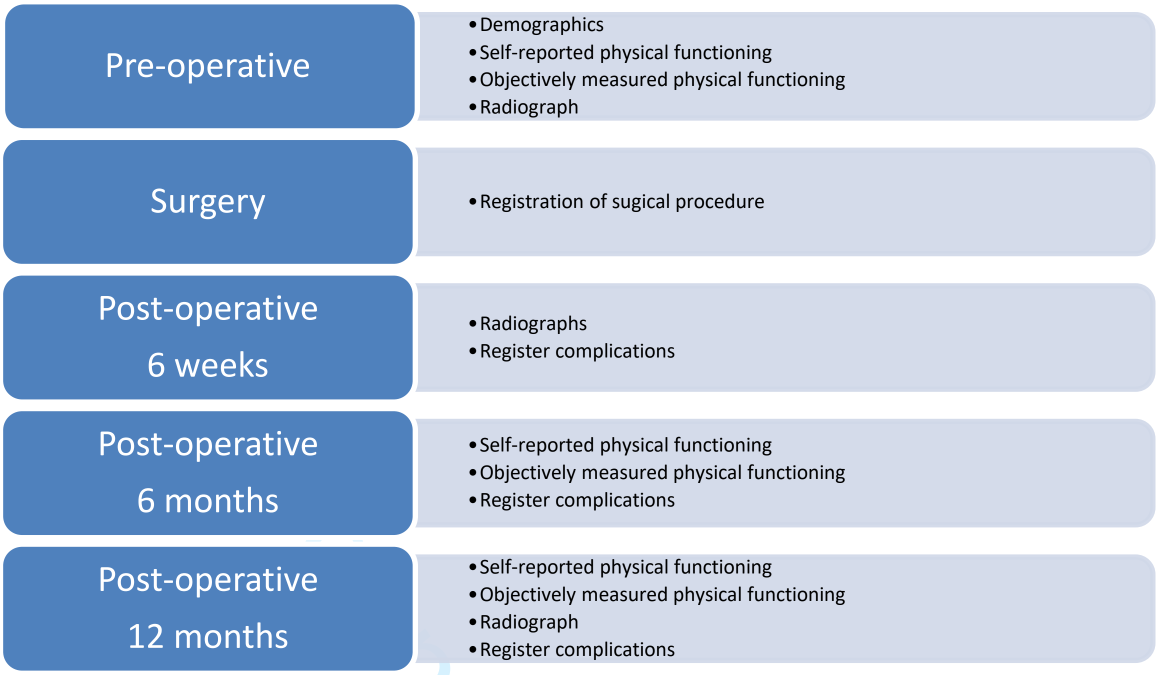

Figure 1. Study procedures

STROBE Statement—Checklist of items that should be included in reports of ***cohort studies***

	Item No	Recommendation	Line
**Title and abstract**	1	(*a*) Indicate the study's design with a commonly used term in the title or the abstract	36
		(*b*) Provide in the abstract an informative and balanced summary of what was done and what was found	36-41
**Introduction**			
Background/rationale	2	Explain the scientific background and rationale for the investigation being reported	60-88
Objectives	3	State specific objectives, including any prespecified hypotheses	89-96
**Methods**			
Study design	4	Present key elements of study design early in the paper	98-104
Setting	5	Describe the setting, locations, and relevant dates, including periods of recruitment, exposure, follow-up, and data collection	99-111 and 228-236
Participants	6	(*a*) Give the eligibility criteria, and the sources and methods of selection of participants. Describe methods of follow-up	114-121
		(*b*) For matched studies, give matching criteria and number of exposed and unexposed	NA
Variables	7	Clearly define all outcomes, exposures, predictors, potential confounders, and effect modifiers. Give diagnostic criteria, if applicable	158-226
Data sources/ measurement	8*	For each variable of interest, give sources of data and details of methods of assessment (measurement). Describe comparability of assessment methods if there is more than one group	158-226
Bias	9	Describe any efforts to address potential sources of bias	100-101, 103-104, 236-237
Study size	10	Explain how the study size was arrived at	238-247
Quantitative variables	11	Explain how quantitative variables were handled in the analyses. If applicable, describe which groupings were chosen and why	249
Statistical methods	12	(*a*) Describe all statistical methods, including those used to control for confounding	250-260
		(*b*) Describe any methods used to examine subgroups and interactions	250-260
		(*c*) Explain how missing data were addressed	260-261
		(*d*) If applicable, explain how loss to follow-up was addressed	NA
		(*e*) Describe any sensitivity analyses	NA
**Results**			
Participants	13*	(a) Report numbers of individuals at each stage of study—eg numbers potentially eligible, examined for eligibility, confirmed eligible, included in the study, completing follow-up, and analysed	NA
		(b) Give reasons for non-participation at each stage	NA
		(c) Consider use of a flow diagram	NA
Descriptive data	14*	(a) Give characteristics of study participants (eg demographic, clinical, social) and information on exposures and potential confounders	NA
		(b) Indicate number of participants with missing data for each variable of interest	NA

For peer review only - http://bmjopen.bmj.com/site/about/guidelines.xhtml

		(c) Summarise follow-up time (eg, average and total amount)	NA
Outcome data	15*	Report numbers of outcome events or summary measures over time	NA
Main results	16	(*a*) Give unadjusted estimates and, if applicable, confounder-adjusted estimates and their precision (eg, 95% confidence interval). Make clear which confounders were adjusted for and why they were included	NA
		(*b*) Report category boundaries when continuous variables were categorized	NA
		(*c*) If relevant, consider translating estimates of relative risk into absolute risk for a meaningful time period	NA
Other analyses	17	Report other analyses done—eg analyses of subgroups and interactions, and sensitivity analyses	NA
**Discussion**			
Key results	18	Summarise key results with reference to study objectives	NA
Limitations	19	Discuss limitations of the study, taking into account sources of potential bias or imprecision. Discuss both direction and magnitude of any potential bias	57
Interpretation	20	Give a cautious overall interpretation of results considering objectives, limitations, multiplicity of analyses, results from similar studies, and other relevant evidence	282-297
Generalisability	21	Discuss the generalisability (external validity) of the study results	298-302
**Other information**			
Funding	22	Give the source of funding and the role of the funders for the present study and, if applicable, for the original study on which the present article is based	NA

*Give information separately for exposed and unexposed groups.

Note: An Explanation and Elaboration article discusses each checklist item and gives methodological background and published examples of transparent reporting. The STROBE checklist is best used in conjunction with this article (freely available on the Web sites of PLoS Medicine at http://www.plosmedicine.org/, Annals of Internal Medicine at http://www.annals.org/, and Epidemiology at http://www.epidem.com/). Information on the STROBE Initiative is available at http://www.strobe-statement.org.

For peer review only

For peer review only - http://bmjopen.bmj.com/site/about/guidelines.xhtml
